# *Prevotella*-dominated dysbiosis and predicted suppression of sulfur biosynthesis pathways associated with halitosis in Korean adults

**DOI:** 10.1128/spectrum.00906-26

**Published:** 2026-09-16

**Authors:** Je-Hyun Eom, Mu-Yeol Cho, Ji-Won Kim, Yunwoo Kim, Seung-Jo Yang, Jiyoung Hwang, Dahye Lee, Hye-Sung Kim, Hanseung Baek, Young-Youn Kim

**Affiliations:** 1 Apple Tree Institute of Biomedical Science, Apple Tree Medical Foundation, Goyang-si, South Korea; 2 Docsmedi Co., Ltd. 656347 , Goyang-si, South Korea; 3 Apple Tree Dental Hospital, Apple Tree Medical Foundation, Goyang-si, South Korea; Nova Southeastern University, Fort Lauderdale, Florida, USA

**Keywords:** halitosis, oral microbiome, *Prevotella*, volatile sulfur compounds, dysbiosis, predicted sulfur metabolism, 16S rRNA sequencing, oral gargle microbiome, Korean adults, microbial biomarkers

## Abstract

Halitosis affects a substantial proportion of adults worldwide, yet the compositional and functional basis of the oral microbiome in halitosis remains incompletely understood, particularly in East Asian populations. We performed 16S rRNA gene amplicon sequencing of oral gargle samples from 45 individuals with halitosis and 45 healthy controls in South Korea, generating approximately 4,600 amplicon sequence variants. Beta diversity analysis revealed significant community-level separation (PERMANOVA, *P* = 0.001) and an elevated Firmicutes/Bacteroidota ratio in halitosis, although alpha diversity did not differ between groups. Because the halitosis group was significantly older than controls, age-adjusted PERMANOVA and age-matched sensitivity analyses confirmed that separation persisted independent of age (age-adjusted *R*² = 0.030, *P* = 0.003). Halitosis-associated microbiomes were enriched for *Prevotella melaninogenica* and *Peptostreptococcus stomatis*, whereas healthy microbiomes were distinguished by *Haemophilus parainfluenzae* and *Veillonella rogosae* (LEfSe). Functional profiling using PICRUSt2 revealed a counterintuitive, predicted suppression of sulfur metabolism in halitosis: pathways for sulfate assimilation, cysteine, and methionine biosynthesis were predicted to be significantly reduced relative to healthy controls. This predicted shift was significantly inversely correlated with directly measured volatile sulfur compounds (H₂S and methyl mercaptan; Spearman ρ = −0.54 and −0.64, both *P* < 0.0001), providing phenotypic support for the predicted signature. These findings suggest healthy oral microbiomes efficiently channel sulfur into anabolic pathways, whereas dysbiotic microbiomes may lose this capacity, allowing volatile sulfur compounds to accumulate. Our study provides a species-resolved, functionally annotated reference framework for halitosis-associated oral dysbiosis in a Korean cohort, and identifies candidate targets for diagnosis and intervention.

**IMPORTANCE:**

Halitosis (bad breath) affects up to 30% of adults worldwide and is caused by volatile sulfur compounds (VSCs) produced by oral bacteria, yet the underlying microbial mechanisms remain poorly understood. Our study of 90 Korean adults reveals that halitosis is associated not only with an overgrowth of *Prevotella* species but also with a predicted, paradoxical suppression of the microbial community’s capacity to assimilate sulfur into anabolic biosynthetic pathways. This predicted “sulfur assimilation failure” was associated with elevated VSC accumulation, supporting a reframing of halitosis as a community-level metabolic dysfunction rather than simple bacterial overproduction. These findings have direct clinical implications: the identified *Prevotella*-dominated dysbiosis and associated sulfur pathway signatures represent candidate biomarkers for objective halitosis diagnosis, and the restoration of microbial sulfur-channeling capacity offers a rational target for probiotic and prebiotic interventions. This work also contributes a well-characterized oral microbiome reference data set from an underrepresented East Asian population.

## INTRODUCTION

Halitosis, the perception of malodorous breath, affects an estimated 20%–30% of the global adult population and constitutes a significant source of social and psychological burden (1). The etiology of halitosis is predominantly oral in origin, with volatile sulfur compounds (VSCs)—principally hydrogen sulfide (H₂S), methyl mercaptan (CH₃SH), and dimethyl sulfide—accounting for the majority of malodor (2). These compounds are generated through microbial proteolysis and catabolism of sulfur-containing amino acids derived from saliva, gingival crevicular fluid, and desquamated epithelial cells on the tongue dorsum (3).

The oral cavity harbors one of the most diverse and densely colonized microbial ecosystems in the human body, comprising over 700 bacterial species (4). The tongue dorsum, with its complex papillary architecture and anaerobic microenvironments, serves as the principal reservoir for VSC-producing bacteria and is widely regarded as the primary anatomical site of halitosis-associated dysbiosis (5). Culture-independent 16S ribosomal RNA (rRNA) gene sequencing has substantially advanced our understanding of the halitosis microbiome, identifying taxa such as *Fusobacterium nucleatum*, *Prevotella melaninogenica*, *Peptostreptococcus stomatis*, and *Solobacterium moorei* as consistent contributors to oral malodor (6). Conversely, genera including *Haemophilus*, *Veillonella*, and *Streptococcus* have been repeatedly associated with healthy breath in adults (7).

Despite this progress, several critical gaps remain. First, the functional consequences of compositional dysbiosis have received limited attention. The prevailing model of halitosis pathogenesis is largely taxon-centric, attributing malodor to the enrichment of VSC-producing bacteria. However, this framework does not address how community-level metabolic networks are reorganized in halitosis or whether dysbiosis involves broader disruption of sulfur metabolism beyond the production of volatile end products. In particular, whether the oral microbial community channels sulfur into anabolic biosynthetic pathways—such as cysteine and methionine biosynthesis—or allows its release as volatile compounds represents a question of community metabolic regulation that has not been systematically addressed. Second, most prior studies have characterized halitosis-associated microbiomes at the genus level, leaving species-level biomarker resolution incomplete. Given that ecologically and metabolically distinct species co-occur within the same genus—particularly within *Prevotella* and *Veillonella*—genus-level profiling may obscure biologically meaningful differences in community functional potential. This concern is not merely theoretical: prior work has shown that closely related *Prevotella* species can display divergent ecological associations even within the same genus-level classification (4, 8), and comparable heterogeneity has been documented within other halitosis-relevant genera such as *Veillonella*, where individual species differ in their metabolic contribution to volatile sulfur compound production despite shared genus-level taxonomy (7). Resolving these differences at the species level is thus a prerequisite for identifying biologically and clinically meaningful biomarkers, rather than genus-level signals that may obscure the specific taxa driving dysbiosis. Third, the majority of published cohort studies originate from European or North American populations, and population-specific microbiome variation has been documented across diverse geographic and ethnic groups (9). Whether the compositional and functional signatures of halitosis generalize to East Asian adults has not been systematically evaluated (10).

Here, we present a 16S rRNA amplicon sequencing study of the oral microbiome in a Korean adult cohort comprising 45 individuals with halitosis and 45 healthy controls, using oral gargle samples as a non-invasive and clinically practical sampling approach. Using amplicon sequence variant (ASV)-based resolution, we characterize community-level differences in diversity, taxonomic composition, and species-level biomarkers through linear discriminant analysis (LDA) effect size (LEfSe). We further apply phylogenetic investigation of communities by reconstruction of unobserved states (PICRUSt2) to predict MetaCyc metabolic pathway activity and interrogate sulfur metabolism in particular. Our findings reveal a *Prevotella*-dominated dysbiosis at the species level and, counterintuitively, a broad predicted suppression of sulfur biosynthesis pathways in halitosis relative to healthy controls—a pattern that reframes halitosis from a taxon-centric model of volatile sulfur compound overproduction to a community-level model of sulfur assimilation failure, in which dysbiotic communities may lose part of the metabolic capacity to channel sulfur into biosynthetic pathways, potentially resulting in the net accumulation and release of volatile sulfur compounds.

## RESULTS

The demographic and clinical characteristics of all participants are summarized in Table 1. The halitosis and healthy control groups did not differ significantly in sex distribution (*P =* 0.582) but differed significantly in age (*P <* 0.001) and in all volatile sulfur compound measurements (*P <* 0.001 for H₂S and MM).

**TABLE 1:** Demographic and clinical characteristics of participants^*a*^^,*d*^

Characteristic	Halitosis (*n* = 45)	Normal (*n* = 45)	*P* value
*n*	45	45	–^*e*^
Sex, *n* (%)			0.582^*c*^
Female	39 (86.7%)	37 (82.2%)	
Male	6 (13.3%)	8 (17.8%)	
Age (years), mean ± SD	48.2 ± 11.2	27.8 ± 6.2	<0.001^*b*^
H₂S (ppb), median (IQR)	358.1 (2.0–881.0)	0.0 (0.0–0.0)	<0.001^*b*^
MM (ppb), median (IQR)	90.0 (33.8–449.6)	0.0 (0.0–0.0)	<0.001^*b*^

^*a*^ Data are presented as mean ± standard deviation (SD) or median (interquartile range, IQR) as appropriate.

^*b*^ Wilcoxon rank-sum test.

^*c*^ Chi-squared test.

^*d*^ H₂S, hydrogen sulfide; MM, methyl mercaptan; IQR, interquartile range.

^*e*^ –, not applicable.

### Alpha diversity does not differ between halitosis and healthy groups

To assess within-sample microbial richness and diversity, we compared four alpha diversity indices—observed species, Shannon index, Simpson index, and Faith’s phylogenetic diversity (PD)—between the halitosis (*n =* 45) and healthy control (*n =* 45) groups. Sequencing depth was sufficient and comparable across all samples, as confirmed by rarefaction curve analysis (Fig. S1). None of the four indices showed a statistically significant difference between groups (Wilcoxon rank-sum test with Benjamini–Hochberg correction; observed species: adj. *P =* 0.089; Shannon index: adj. *P =* 0.773; Simpson index: adj. *P =* 0.773; Faith’s PD: adj. *P =* 0.773; Fig. 1). These results indicate that halitosis is not associated with a reduction in overall species richness or phylogenetic diversity, suggesting that the oral microbial community structure in halitosis is characterized by compositional shifts rather than a loss of diversity *per se*.

**Fig 1:**
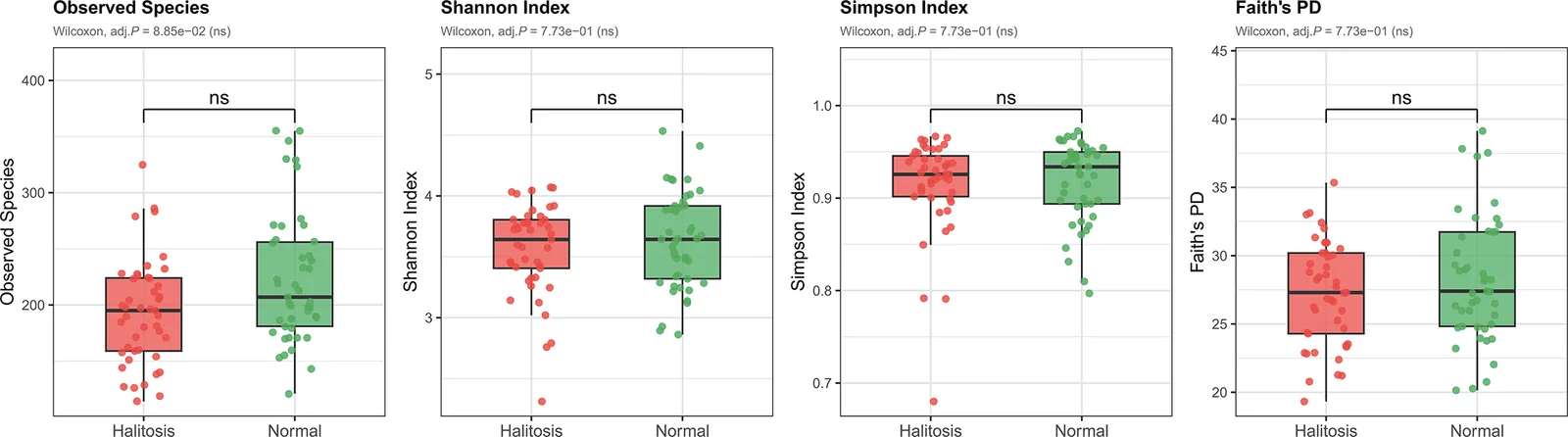
Alpha diversity comparison between halitosis and healthy control groups. Box plots showing four alpha diversity metrics—observed species richness, Shannon index, Simpson index, and Faith’s phylogenetic diversity—in halitosis (*n =* 45) and healthy control (*n =* 45) groups. Statistical comparisons were performed using the Wilcoxon rank-sum test with Benjamini–Hochberg false discovery rate correction. ns, not significant. Center lines indicate medians; box limits indicate the 25th and 75th percentiles; whiskers extend to 1.5×the interquartile range. Four side-by-side box plots comparing alpha diversity metrics between halitosis and healthy control groups. Panels show observed species richness (adj. *P =* 0.089), Shannon index (adj. *P =* 0.773), Simpson index (adj. *P =* 0.773), and Faith’s phylogenetic diversity (adj. *P =* 0.773). All comparisons are non-significant, indicating no meaningful difference in within-sample diversity between groups.

### Beta diversity reveals significant community-level separation between groups

To evaluate between-sample compositional differences, we performed principal coordinates analysis (PCoA) based on Bray–Curtis dissimilarity. The two groups showed significant separation along the primary axes of variation (PERMANOVA, *P =* 0.001, *R²* = 0.052), with PC1 and PC2 explaining 16.1% and 8.8%, respectively, of the total variance (Fig. 2). Homogeneity of multivariate dispersions was confirmed prior to PERMANOVA interpretation (permutation test, *P =* 0.471; Fig. S2), indicating that the observed separation reflects a true difference in community composition rather than a difference in within-group variability. Although the effect size was modest (*R²* = 0.052), consistent with the known interindividual variability of the oral microbiome, the result demonstrates that halitosis is associated with a reproducible and statistically robust shift in overall microbial community structure. To account for the significant age difference between groups (halitosis: mean 48.2 ± 11.2 years; normal: mean 27.8 ± 6.2 years; Table 1), we performed an age-adjusted PERMANOVA including age as a covariate. Group membership remained a significant and independent predictor of microbial community composition after controlling for age (*R²* = 0.030, *P =* 0.003; Table S1), confirming that the observed beta diversity separation was not attributable to age differences alone. As a further test of this age confound, we examined whether the principal coordinates driving community separation tracked age independently of halitosis status. PC1 and PC2 were both significantly correlated with age (Spearman *ρ* = −0.24, *P* = 0.020, and *ρ* = 0.44, *P* = 1.4 × 10⁻⁵, respectively; Fig. S3), confirming that age contributes to overall community variation, with PC2 showing the stronger association. However, halitosis-associated group separation remained visually evident even among age-overlapping individuals (Fig. S3), indicating that group separation is not solely attributable to the age difference between cohorts.

**Fig 2:**
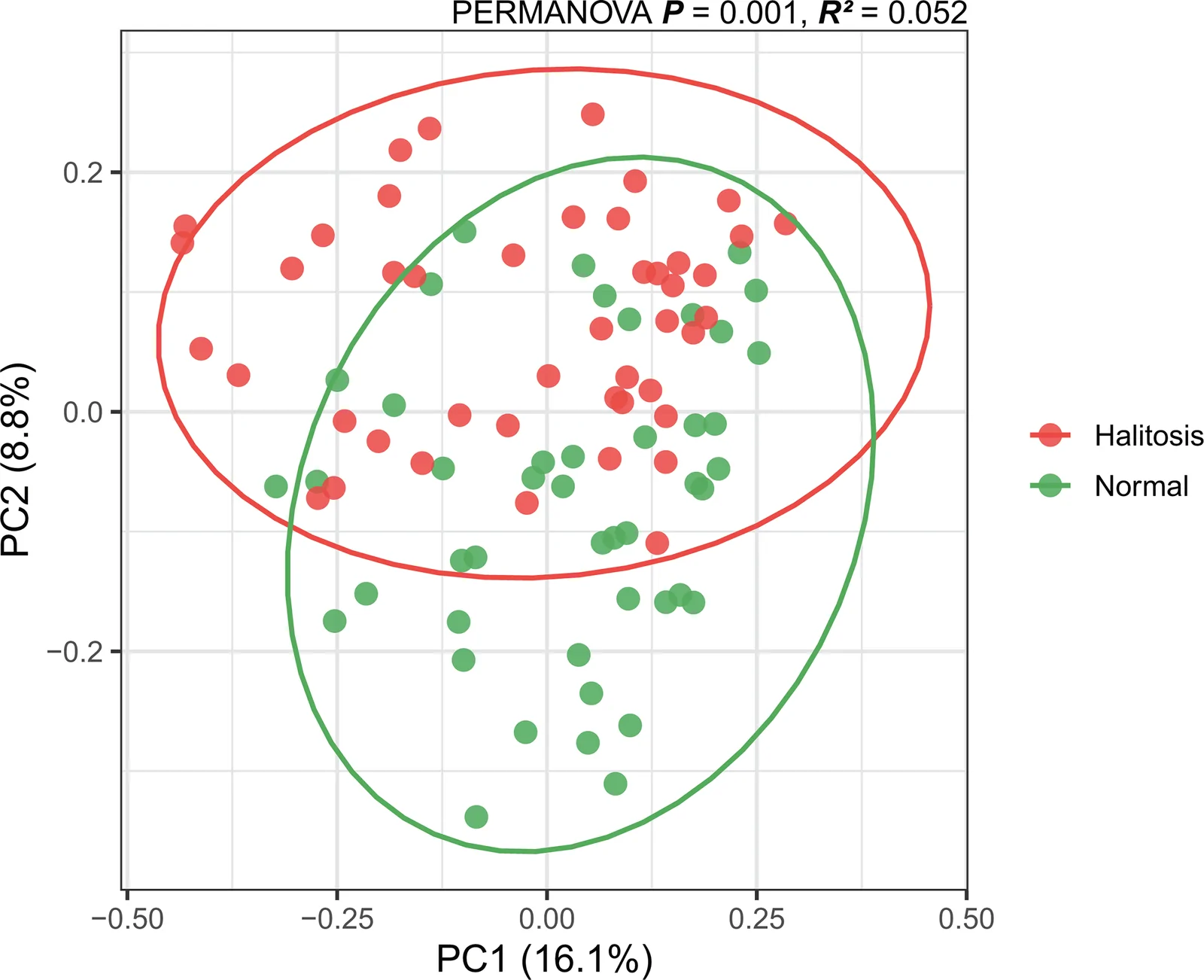
Beta diversity analysis of oral microbial communities. Principal coordinates analysis (PCoA) plot based on Bray–Curtis dissimilarity for halitosis (red, *n =* 45) and healthy control (blue, *n =* 45) groups. Each point represents one individual. The percentage of variance explained by each axis is indicated. Statistical significance of group separation was assessed by PERMANOVA (*P =* 0.001, *R*² = 0.052). Homogeneity of multivariate dispersions was confirmed by permutation test (*P =* 0.471). A scatter plot showing principal coordinates analysis based on Bray–Curtis dissimilarity. each point represents one individual, with halitosis samples and healthy control samples forming partially overlapping but distinct clusters. PC1 explains 16.1% and PC2 explains 8.8% of total variance. The two groups show statistically significant separation (PERMANOVA *P =* 0.001, *R*² =0.052).

To formally test this, we performed a sensitivity analysis in which PERMANOVA was repeated on age-matched subsets of the cohort (Table S2 and Fig. S4). Group separation remained statistically significant across all age-matched windows tested, including a stringent subset restricted to 22–45 years (*n* = 17 halitosis, 44 normal; R² = 0.054, *P* = 0.001) and an intentionally narrow 27–35-year subset with substantially reduced sample size and power (*n* = 5 halitosis, 15 normal; *R*² = 0.090, *P* = 0.018). Together with the age-adjusted PERMANOVA above, these converging analyses indicate that halitosis-associated compositional separation is robust to the age imbalance between groups.

### Genus-level differential abundance identifies key halitosis-associated taxa

To identify differentially abundant genera between groups, we applied the Wilcoxon rank-sum test with Benjamini–Hochberg false discovery rate (FDR) correction. Twenty-four genera showed statistically significant differences in relative abundance (Fig. 3). The overall genus-level community composition is shown in Fig. S5. Among genera enriched in the healthy control group, *Haemophilus* showed the most pronounced reduction in halitosis (FDR = 3.03 × 10⁻⁸), while *Acinetobacter* (FDR = 2.36 × 10⁻¹⁵), *Sphingomonas* (FDR = 1.36 × 10⁻¹⁰), *Selenomonas* (FDR = 3.46 × 10⁻⁵), and *Suttonella* (FDR = 4.30 × 10⁻⁵) were also significantly lower in the halitosis group, indicating their association with a healthy oral microbiome. Conversely, *Prevotella* (FDR = 7.97 × 10⁻⁶), *Prevotella_7* (a SILVA-defined *Prevotella* subclade distinct from the *Prevotella* genus grouping above; FDR = 2.19 × 10⁻⁴), *Peptostreptococcus* (FDR = 4.30 × 10⁻⁵), *Mogibacterium* (FDR = 1.28 × 10⁻⁷), and *AlloPrevotella* (FDR = 1.02 × 10⁻⁴) were significantly enriched in the halitosis group. Notably, *Haemophilus* exhibited the highest mean relative abundance among all genera in the healthy group, underscoring its potential role as a marker of a balanced oral microbiome. The predominance of *Prevotella* and related anaerobic genera in halitosis is consistent with their known capacity for proteolytic activity and volatile sulfur compound production. The Firmicutes/Bacteroidota ratio was also significantly elevated in the halitosis group (Fig. S6), further reflecting the broad phylogenetic shift associated with halitosis-associated dysbiosis.

**Fig 3:**
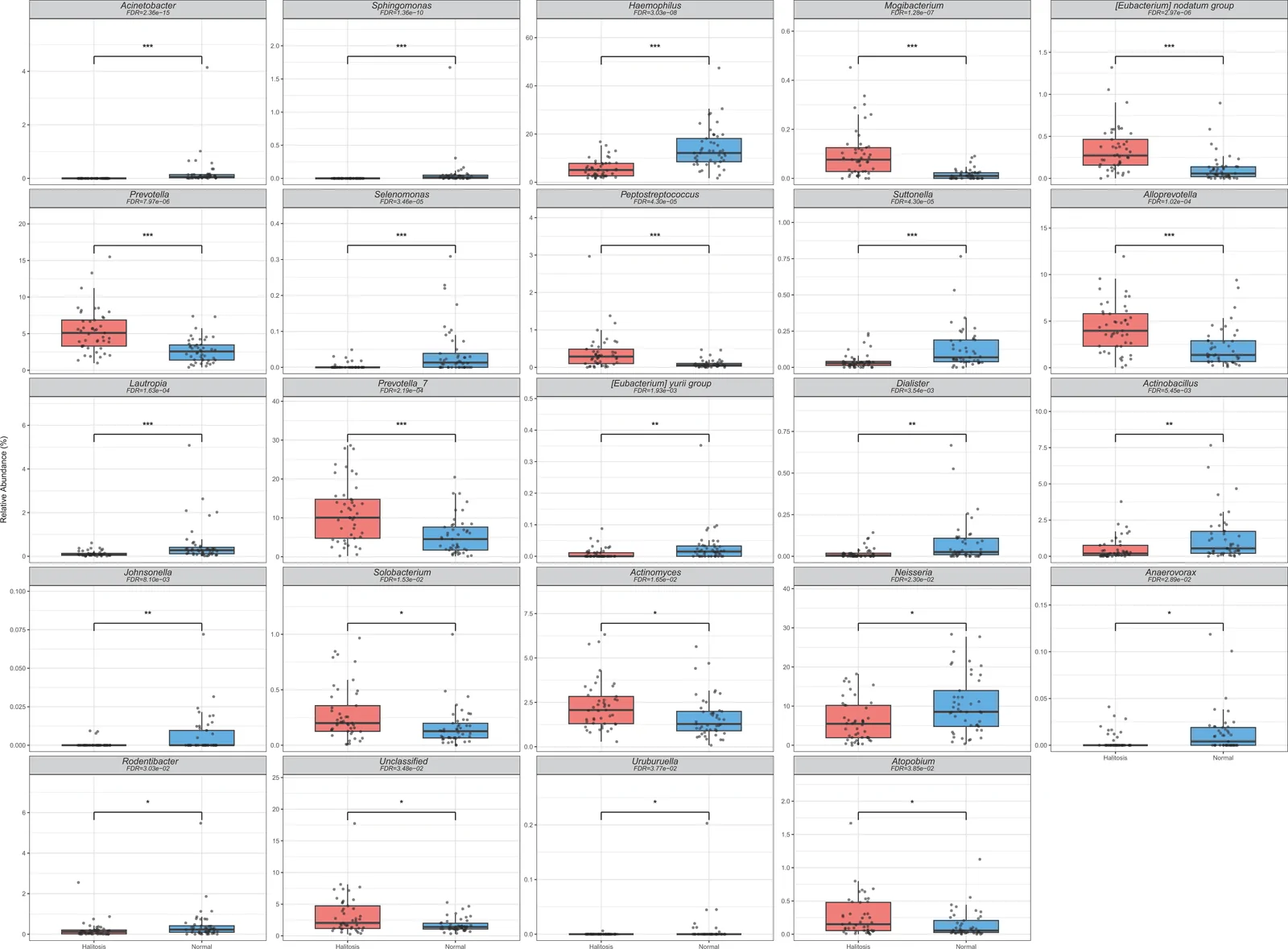
Genus-level differential abundance between halitosis and healthy control groups. Box plots showing the relative abundance (%) of 24 significantly differentially abundant genera, arranged in order of ascending FDR (most statistically significant panels first); genera enriched in the halitosis group and the healthy control group are interspersed throughout rather than grouped by row. Statistical significance was determined by Wilcoxon rank-sum test with Benjamini–Hochberg correction. *, adj. *P <* 0.05; **, adj. *P* < 0.01; ***, adj. *P <* 0.001. FDR values are indicated above each panel. Center lines indicate medians; box limits indicate the 25th and 75th percentiles; whiskers extend to 1.5× the interquartile range. Twenty-four box plots arranged in a grid ordered by ascending FDR from most to least statistically significant. panels for genera enriched in the healthy control group (e.g., *Acinetobacter*, FDR = 2.36 × 10⁻¹⁵; *Sphingomonas; Haemophilus*, FDR = 3.03 × 10⁻⁸; Selenomonas; Suttonella) are interspersed with panels for genera enriched in the halitosis group (e.g., *Mogibacterium; Prevotella; Peptostreptococcus; Alloprevotella; Prevotella_7*) according to statistical significance rather than direction. All differences are statistically significant after multiple testing correction (FDR < 0.05).

### Species-level analysis reveals distinct compositional signatures and core microbiome differences

To achieve higher taxonomic resolution, we performed species-level differential abundance analysis and core microbiome profiling. A heatmap of Z-score-normalized relative abundances for all 30 differentially abundant species confirmed clear group-level separation (Fig. 4A). Species enriched in the halitosis group included *Prevotella* sp., *Peptostreptococcus stomatis*, *Prevotella nanceiensis*, *AlloPrevotella* sp., *[Eubacterium] nodatum* group sp., and *Mogibacterium* sp., whereas *Veillonella rogosae*, *Haemophilus* sp., *Lautropia* sp., *Sphingomonas* sp., and *Acinetobacter* sp. were predominantly associated with the healthy group. Species-level stacked bar composition and mean relative abundances of the top 25 species are presented in Fig. S7 and S8, respectively, and differential abundance box plots for individual species are shown in Fig. S9.

**Fig 4:**
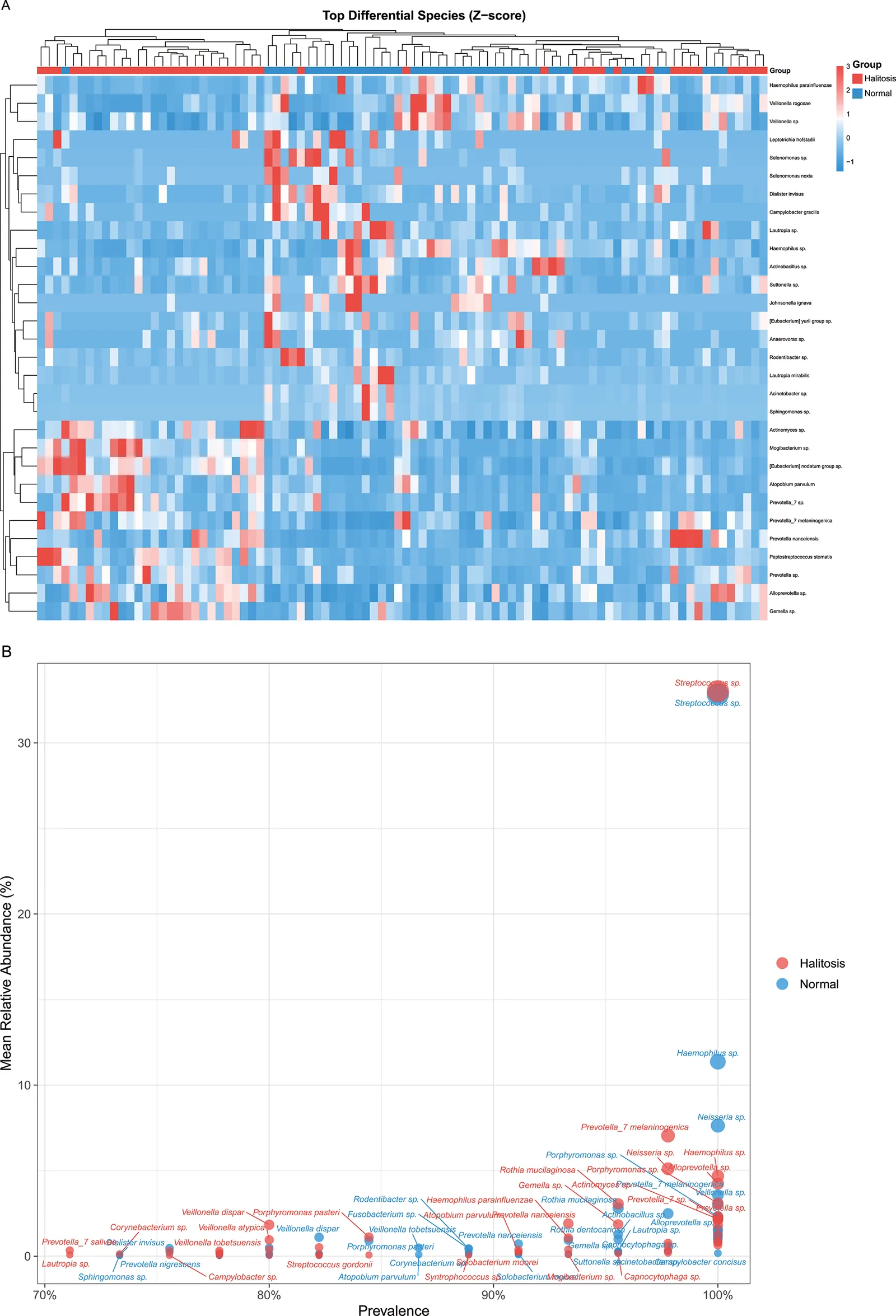
Species-level differential abundance and core microbiome composition. (**A**) Heatmap showing Z-score-normalized relative abundances of the 30 differentially abundant species across all samples. Red and blue column bars indicate halitosis and healthy control groups, respectively. Species are ordered by their differential abundance pattern. Color scale represents the Z-score (red, high relative abundance; blue, low relative abundance). (**B**) Bubble plot of core species (present in ≥70% of samples within each group) showing mean relative abundance (%) on the y-axis and prevalence on the x-axis. Bubble size is proportional to the mean relative abundance. Red bubbles, halitosis group; blue bubbles, healthy control group. Species labels are shown for all core members. Panel A shows a heatmap of Z-score-normalized relative abundances for 30 differentially abundant species across 90 samples. Halitosis samples (left columns) show higher abundances of *prevotella* species and *Peptostreptococcus stomatis*, while healthy control samples (right columns) show higher abundances of *Haemophilus*, *Veillonella rogosae*, and *Acinetobacter* species, forming visually distinct color patterns between groups. Panel B shows a bubble plot of core microbiome species at 70% prevalence threshold. *Streptococcus* sp. is the most abundant core species in both groups. *Haemophilus* sp. shows higher mean relative abundance in healthy controls, while *Prevotella_7 melaninogenica* is more abundant in the halitosis group.

Core microbiome analysis, defined as species present in at least 70% of samples within each group, identified marked differences in both prevalence and mean relative abundance (Fig. 4B). *Streptococcus* sp. was the most abundant core species in both groups, present at near-universal prevalence (>99%) with comparable mean relative abundances exceeding 30%. However, *Haemophilus* sp. was a prominent core member in the healthy group with a mean relative abundance of approximately 11%, while it was comparatively reduced in the halitosis group. Conversely, *Prevotella_7 melaninogenica* exhibited substantially higher mean relative abundance in the halitosis core microbiome. Several species including *P. nanceiensis*, *Solobacterium moorei*, *Atopobium parvulum*, and *AlloPrevotella* sp. were shared core members across both groups but differed in their relative abundances, suggesting that halitosis-associated dysbiosis involves quantitative shifts within a largely shared species pool rather than wholesale replacement of community members. In total, 30 species showed statistically significant differential abundance between groups (Wilcoxon rank-sum test, FDR < 0.05).

### LEfSe identifies species-level biomarkers discriminating halitosis from healthy controls

To identify species that most robustly discriminate between groups, we applied linear discriminant analysis effect size (LEfSe) with an LDA score threshold of 2.0 (Fig. 5). Six species were significantly enriched in the halitosis group: *P. melaninogenica* (LDA = 5.34), *P. nanceiensis* (LDA = 4.82), *Prevotella histicola* (LDA = 4.63), *Peptostreptococcus stomatis* (LDA = 4.12), *Prevotella salivae* (LDA = 4.03), and *Atopobium parvulum* (LDA = 3.89). The dominance of multiple *Prevotella* species among the top halitosis-enriched taxa underscores the central role of this genus in oral dysbiosis associated with malodor.

**Fig 5:**
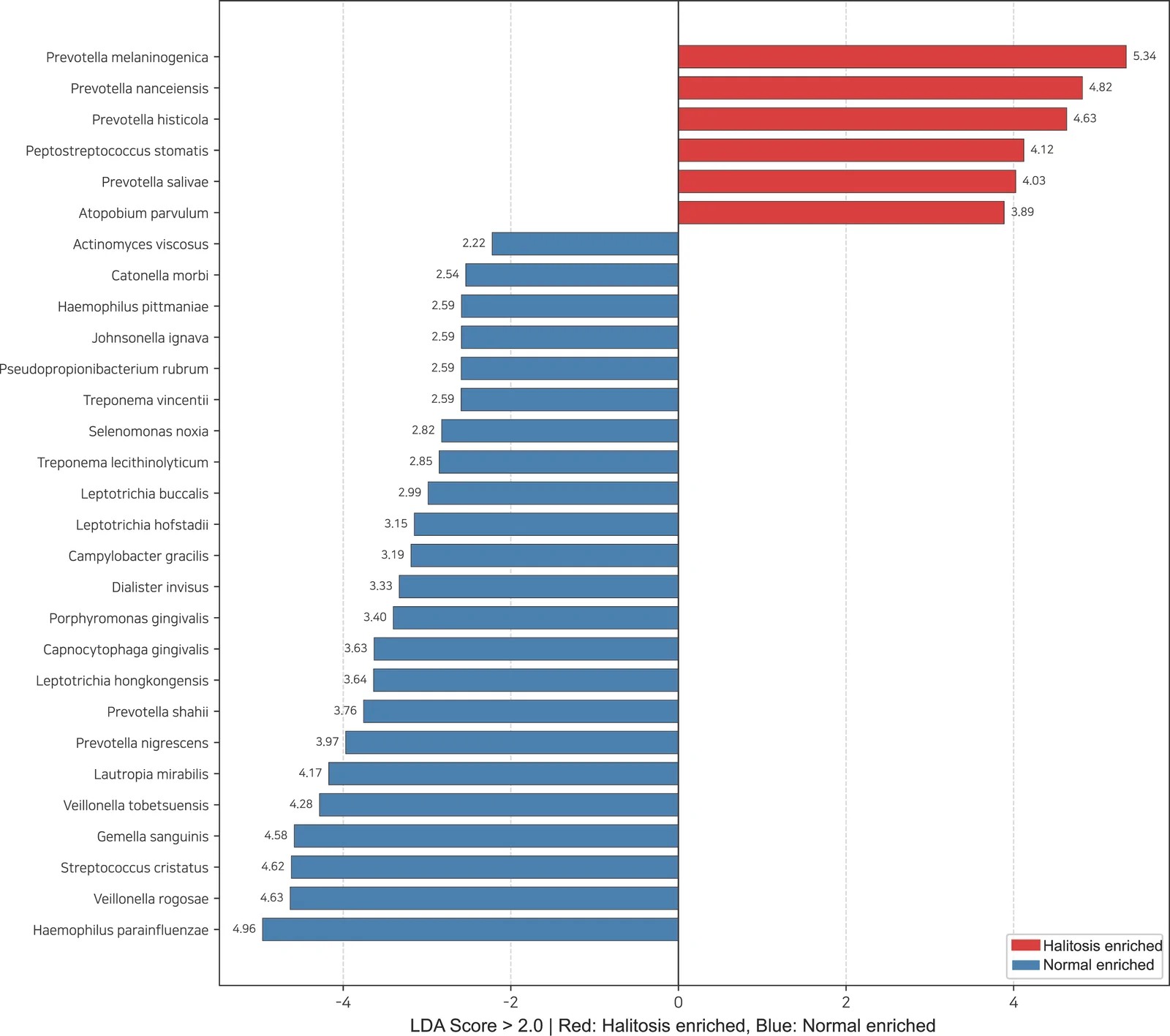
LEfSe analysis of species-level differential taxa. Horizontal bar chart showing species with a linear discriminant analysis (LDA) score greater than 2.0. Red bars indicate species enriched in the halitosis group; blue bars indicate species enriched in the healthy control group. LDA scores are shown at the end of each bar. Taxa are ranked by absolute LDA score within each group. A horizontal bar chart showing LDA scores for 29 discriminant species. six species with red bars are enriched in the halitosis group, with *Prevotella melaninogenica* having the highest score (LDA = 5.34), followed by *Prevotella nanceiensis* (4.82), *Prevotella histicola* (4.63), *Peptostreptococcus stomatis* (4.12), *Prevotella salivae* (4.03), and *Atopobium parvulum* (3.89). Twenty-three species with blue bars are enriched in the healthy control group, with *Haemophilus parainfluenzae* having the highest score (LDA = 4.96).

Twenty-three species were significantly enriched in the healthy control group, with *Haemophilus parainfluenzae* showing the highest LDA score (4.96), followed by *Veillonella rogosae* (4.63), *Streptococcus cristatus* (4.62), *Gemella sanguinis* (4.58), and *Veillonella tobetsuensis* (4.28). Additional healthy-associated species included *Lautropia mirabilis* (4.17), *Prevotella nigrescens* (3.97), *Prevotella shahii* (3.76), *Leptotrichia hongkongensis* (3.64), and *Capnocytophaga gingivalis* (3.63), among others. The substantially greater number of health-associated discriminant species relative to halitosis-enriched species suggests that a diverse and balanced oral community characterizes the healthy state, while halitosis is driven by the outgrowth of a limited set of anaerobic proteolytic taxa.

### Age-adjusted differential abundance analysis confirms consistent direction of effect and retains significance for the majority of taxa

Because genus- and species-level differential abundance testing above did not account for the age difference between groups, we repeated these analyses using an age-adjusted linear model (arcsine-square-root-transformed relative abundance ~ group + age). Of the taxa that had been significant in the unadjusted Wilcoxon analysis (24 genera and 30 species at FDR < 0.05; see Genus-level and Species-level sections above; Table S3), the direction of the halitosis-associated effect was preserved for 100% of taxa (24/24 genera and 30/30 species) in the age-adjusted model, indicating no reversal of the reported associations. Formal statistical significance after age adjustment and Benjamini–Hochberg correction (applied within this targeted set of previously significant taxa) was retained for the majority of taxa: 15 of the 24 genera and 18 of the 30 species remained significant at FDR < 0.05 (Table S3), while the remainder showed attenuated but directionally consistent associations. This partial attenuation most likely reflects reduced statistical power arising from collinearity between age and halitosis status in this cohort (halitosis: mean 48.2 years; normal: mean 27.8 years) rather than a true absence of effect, given that the direction of every association was preserved. Among taxa central to the compositional signature reported above, *Peptostreptococcus stomatis*, *Veillonella rogosae*, and *Prevotella_7 melaninogenica* (species level) retained formal significance after age adjustment, whereas *Prevotella_7* (genus level) and *Haemophilus parainfluenzae* showed attenuated but directionally consistent associations. These results indicate that, in contrast to the community-level PERMANOVA and age-matched sensitivity analyses described above, individual taxon-level significance testing was somewhat more sensitive to the age imbalance between groups for a subset of taxa, underscoring the importance of age-matched recruitment in future studies to establish fully independent taxon-level inference for the entire signature.

### Functional profiling reveals predicted broad suppression of metabolic pathways in halitosis, with a paradoxical reduction in sulfur biosynthesis

To investigate functional differences between groups, we predicted MetaCyc pathway abundances using PICRUSt2. Prediction reliability, assessed using the weighted Nearest Sequenced Taxon Index (NSTI), was uniformly high across the data set (14 of 4,598 ASVs excluded for exceeding the recommended NSTI cutoff of 2.0) and did not differ significantly between groups (halitosis: mean NSTI = 0.038 ± 0.011; normal: 0.034 ± 0.005; Wilcoxon rank-sum test, *P* = 0.180; Table S4), indicating comparable prediction confidence across both study groups. All statistically significant pathways (*P* < 0.05) with the largest log_2_ fold-change magnitude were higher in the healthy control group relative to halitosis (Fig. 6A). Among these 30 pathways, the majority carried FDR < 0.05, indicating robust differences after multiple testing correction. Pathways with the largest log_2_ fold changes included unsaturated and even-numbered fatty acid β-oxidation, oleate β-oxidation, phytate degradation I, and PWY-8020, suggesting broad impairment of diverse catabolic and anabolic capacities in the halitosis microbiome. The top 40 differential pathways across all samples are visualized as a heatmap in Fig. S10.

**Fig 6:**
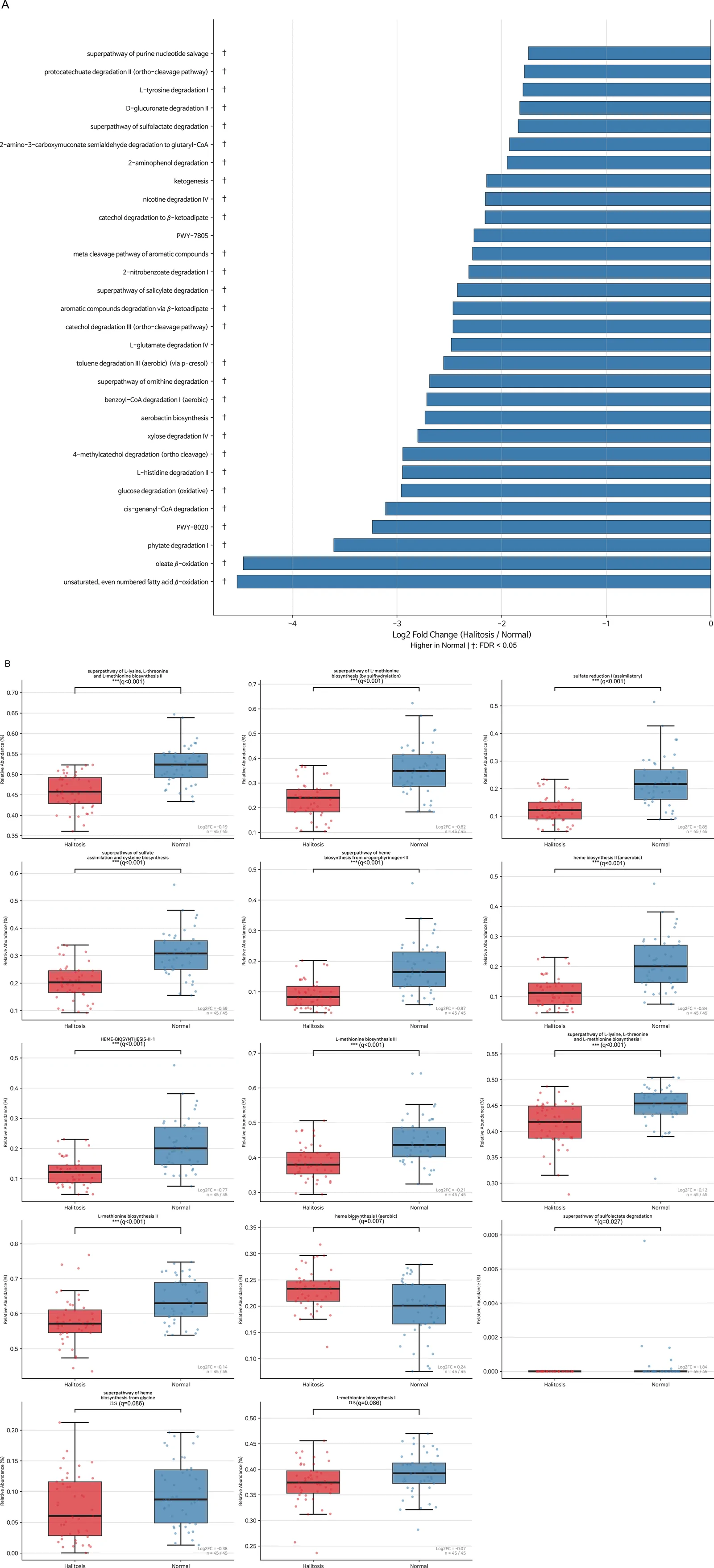
Functional pathway analysis using PICRUSt2. (**A**) Horizontal bar chart of the 30 pathways with the largest fold-change magnitude among pathways reaching nominal significance (*P* < 0.05), all higher in the healthy control group. All bars represent log_2_ fold change of halitosis relative to normal (negative values indicate depletion in halitosis). †, FDR < 0.05 after Benjamini–Hochberg correction. (**B**) Box plots showing predicted relative abundances (%) of 14 sulfur metabolism-related MetaCyc pathways in halitosis (red, *n =* 45) and healthy control (blue, *n =* 45) groups. Log_2_ fold change (Log_2_FC) values and sample sizes are indicated within each panel. Statistical significance was assessed by Wilcoxon rank-sum test with Benjamini–Hochberg correction. *, *q* < 0.05; **, *q* < 0.01; ***, *q <* 0.001. Center lines indicate medians; box limits indicate the 25th and 75th percentiles; whiskers extend to 1.5× the interquartile range; individual data points are overlaid. Panel **A** shows a horizontal bar chart of the 30 MetaCyc pathways with the largest fold-change magnitude among those reaching nominal significance, all higher in healthy controls. All bars extend to the left (negative log2 fold change), indicating depletion in halitosis. Pathways with the largest fold changes include unsaturated fatty acid beta-oxidation and oleate beta-oxidation. The majority of pathways are marked with a dagger symbol indicating FDR < 0.05. Panel B shows 14 box plots of sulfur metabolism-related pathways. thirteen of the 14 pathways show lower predicted relative abundances in the halitosis group compared to healthy controls, including sulfate assimilation and cysteine biosynthesis (Log2FC = −0.59, *q* < 0.001) and sulfolactate degradation (Log2FC = −1.84, *q* = 0.027). One pathway, heme biosynthesis I (aerobic), shows the opposite pattern, with higher predicted abundance in halitosis (Log2FC = 0.24, *q* = 0.007).

Strikingly, detailed examination of sulfur metabolism pathways revealed a predicted suppression across the majority of measured pathways in the halitosis group, with one notable exception (Fig. 6B). The superpathway of L-lysine, L-threonine, and L-methionine biosynthesis II (Log_2_FC = −0.19, *q <* 0.001), superpathway of L-methionine biosynthesis by sulfhydrylation (Log_2_FC = −0.62, *q <* 0.001), and sulfate reduction I assimilatory pathway (Log_2_FC = −0.85, *q <* 0.001) were all significantly reduced in halitosis. Similarly, the superpathway of sulfate assimilation and cysteine biosynthesis (Log_2_FC = −0.59, *q <* 0.001), heme biosynthesis pathways, L-methionine biosynthesis II (Log2FC = −0.14, *q <* 0.001), and superpathway of sulfolactate degradation (Log2FC = −1.84, *q =* 0.027) were consistently lower in halitosis. In contrast, heme biosynthesis I (aerobic) was the sole pathway among the 14 examined that was significantly higher in halitosis (Log_2_FC = 0.24, *q* = 0.007), indicating that not all heme-related biosynthetic routes are uniformly affected. These findings indicate that rather than an upregulation of sulfur compound production, the halitosis microbiome may be characterized by a failure to efficiently assimilate sulfur into biosynthetic pathways, potentially allowing volatile sulfur compounds to accumulate and be released into the oral environment.

### Predicted sulfur pathway abundance correlates with directly measured volatile sulfur compounds

To determine whether the PICRUSt2-predicted sulfur assimilation deficit reflects the actual malodor phenotype rather than genomic potential alone, we tested the association between predicted sulfur pathway abundance and the volatile sulfur compound concentrations directly measured in all 90 participants at the time of sample collection (Fig. 7). A composite score summarizing a subset of eight core pathways—selected from the 14 sulfur metabolism-related MetaCyc pathways shown in Fig. 6B based on their direct annotation to sulfate assimilation, cysteine/methionine biosynthesis, or heme biosynthesis—was significantly inversely correlated with both measured hydrogen sulfide (Spearman *ρ* = −0.54, *P* = 4.7 × 10⁻⁸) and methyl mercaptan (*ρ* = −0.64, *P* = 1.0 × 10⁻¹¹) concentrations. These correlations remained statistically significant after adjusting for age (partial Spearman *ρ* = −0.29, *P* = 0.007 for H₂S; *ρ* = −0.40, *P* < 0.001 for methyl mercaptan; Table S5), indicating that the association between predicted sulfur pathway abundance and measured malodor is not attributable to the age difference between groups. These findings provide independent phenotypic evidence that the predicted sulfur assimilation deficit is associated with the directly measured clinical phenotype.

**Fig 7:**
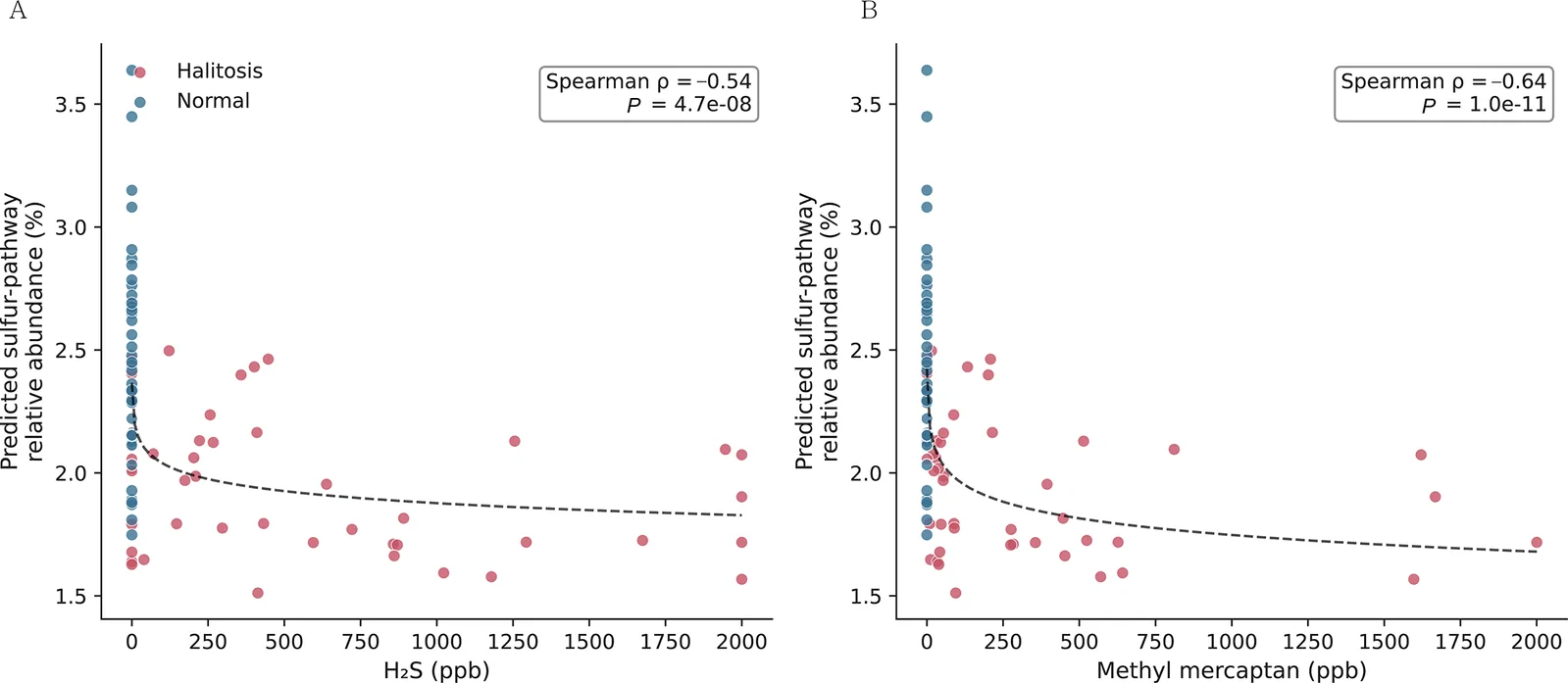
Predicted sulfur pathway abundance correlates with directly measured volatile sulfur compounds. Scatter plots showing the relationship between PICRUSt2-predicted sulfur pathway relative abundance (composite score derived from eight core sulfate assimilation, cysteine/methionine biosynthesis, and heme biosynthesis pathways) and directly measured volatile sulfur compound concentrations in all 90 participants. Halitosis samples are shown in red/pink (*n* = 45); healthy control samples are shown in blue (*n* = 45). Dashed lines indicate the fitted trend. (**A**) Predicted sulfur pathway abundance versus hydrogen sulfide (H₂S) concentration (Spearman *ρ* = −0.54, *P* = 4.7 × 10⁻⁸). (**B**) Predicted sulfur pathway abundance versus methyl mercaptan concentration (Spearman *ρ* = −0.64, *P* = 1.0 × 10⁻¹¹). Two scatter plots showing an inverse relationship between PICRUSt2-predicted sulfur pathway abundance and measured volatile sulfur compounds across 90 participants. In both panels, healthy control samples (blue) cluster near zero VSC concentration with predicted sulfur pathway abundance ranging broadly from about 1.5% to 3.6%, while halitosis samples (red) are distributed across a wide range of VSC concentrations (up to approximately 2,000 ppb) and predominantly show lower predicted sulfur pathway abundance, mostly between 1.5% and 2.5%. Panel A plots predicted abundance against hydrogen sulfide concentration (Spearman *ρ* = −0.54, *P* < 0.0001); Panel B plots predicted abundance against methyl mercaptan concentration (Spearman *ρ* = −0.64, *P* < 0.0001). A dashed trend line in each panel illustrates the overall inverse association.

### Post-hoc power analysis confirms adequate statistical power

Because closed-form power calculations are not available for permutation-based multivariate tests, we performed an empirical, subsampling-based post-hoc power analysis using the observed effect sizes in this data set (Table S6 and Fig. S11). At the community level, 80% power was achieved with as few as nine individuals per group based on the observed PERMANOVA effect size (*R*² = 0.066), and power was effectively saturated (100%) at the actual cohort size of 45 individuals per group. For representative species-level biomarkers spanning a range of effect sizes, 80% power was achieved between 25 and 35 individuals per group (*Prevotella_7 melaninogenica*: *n* = 25; *Haemophilus parainfluenzae*: *n* = 30; *Atopobium parvulum*: *n* = 35), with all three reaching 100% power at the actual sample size. These results confirm that the present cohort was adequately powered for both the community-level and species-level findings reported above.

## DISCUSSION

This study provides a species-resolved and functionally characterized portrait of the oral microbiome in halitosis, drawing on a well-powered Korean adult cohort of 90 individuals. Our principal findings are fourfold: (i) halitosis is associated with significant beta diversity separation without loss of alpha diversity, indicating community compositional reorganization rather than diversity depletion; (ii) *Prevotella*-dominated dysbiosis, characterized by the co-enrichment of multiple *Prevotella* species alongside *Peptostreptococcus stomatis* and *Atopobium parvulum*, characterizes the halitosis microbiome at the species level; (iii*) Haemophilus parainfluenzae*, *Veillonella rogosae*, and *Streptococcus cristatus* consistently characterize the healthy oral microbiome; and (iv) counterintuitively, sulfur assimilation and biosynthesis pathways are predicted to be broadly suppressed in halitosis rather than upregulated, reframing the mechanistic basis of volatile sulfur compound accumulation from a taxon-centric overproduction model to a systems-level model of community metabolic dysfunction. These findings extend prior genus-level characterizations of the halitosis microbiome, most of which have not resolved compositional signatures below the genus level or examined the functional consequences of dysbiosis (6). By combining amplicon sequence variant-based species resolution with PICRUSt2 functional prediction, this study distinguishes ecologically and metabolically distinct species within genera such as *Prevotella*—associations that would otherwise be obscured by genus-level aggregation—and links this compositional shift to a specific, testable functional hypothesis regarding community-level sulfur metabolism.

The absence of significant alpha diversity differences between groups is a recurrent and biologically meaningful finding in oral microbiome research. Several prior studies examining halitosis-associated communities have similarly reported unchanged or only marginally reduced diversity indices (8), in contrast to conditions such as periodontitis, where alpha diversity changes are more pronounced (11, 12). Our results extend this pattern to a Korean adult population and, crucially, demonstrate that the significant beta diversity separation (PERMANOVA *R*² = 0.052, *P =* 0.001) with confirmed dispersion homogeneity reflects genuine compositional divergence. The modest *R*² value is consistent with the substantial interindividual variation inherent to the oral microbiome and should not be interpreted as a weak effect; rather, it reflects the stochastic nature of community assembly superimposed on a reproducible dysbiotic signal (7). This pattern—preserved diversity with altered composition—parallels findings in other human-associated microbiome disorders, including irritable bowel syndrome and bacterial vaginosis, where community-level reorganization rather than species loss underlies functional disruption (13).

The enrichment of multiple *Prevotella* species in halitosis—*P. melaninogenica*, *P. nanceiensis*, *P. histicola*, and *P. salivae*—consolidates and extends findings from prior 16S rRNA and metagenomic studies. *P*. melaninogenica has been identified as a key halitosis biomarker in prior LEfSe analyses (8), and its highest LDA score in our data set (5.34) corroborates this designation in an independent East Asian cohort. The additional identification of *P. nanceiensis* (LDA = 4.82) and *P. histicola* (LDA = 4.63) as strong halitosis discriminants represents a species-level refinement beyond what genus-level analyses can provide. These species possess well-characterized proteolytic and amino acid catabolic capabilities and are capable of producing H₂S and methyl mercaptan from cysteine and methionine substrates (14). The co-enrichment of *Peptostreptococcus stomatis* and *Atopobium parvulum*—both established volatile sulfur compound producers—further reinforces the anaerobic proteolytic character of the halitosis community (15).

On the healthy side, *Haemophilus parainfluenzae* emerged as the most discriminant species (LDA = 4.96), consistent with prior reports of its depletion in dysbiotic oral conditions (16). As a facultative anaerobe with limited proteolytic activity, *H. parainfluenzae* may contribute to a competitive microenvironment that suppresses obligate anaerobes, and its high prevalence and abundance in healthy controls position it as a putative indicator of oral homeostasis. The identification of *Veillonella rogosae*, *Streptococcus cristatus*, and *Gemella sanguinis* as healthy-associated taxa is also biologically coherent: *Veillonella* species are primary lactate consumers that produce short-chain fatty acids without volatile sulfur compound generation (17), while *Streptococcus cristatus* has been reported to inhibit the colonization of periodontal pathogens through arginine deiminase activity (18). Together, these species define a functionally coherent module—facultative anaerobes and lactate-utilizing commensals—whose collective presence may maintain redox and metabolic conditions unfavorable for obligate anaerobic proteolytic taxa.

The most conceptually significant finding of this study is the paradoxical predicted suppression of sulfur metabolism in the halitosis microbiome. The conventional framework holds that halitosis results from the enrichment of volatile sulfur compound-producing bacteria that actively catabolize sulfur-containing compounds. Our PICRUSt2 results challenge this model by demonstrating that the predicted capacity for sulfate assimilation, cysteine biosynthesis, methionine biosynthesis, and sulfolactate degradation is lower in halitosis than in healthy controls for the large majority of pathways examined (13 of 14), with a single exception—heme biosynthesis I (aerobic)—showing the opposite pattern. We propose that this pattern reflects a fundamental difference in how the two communities partition sulfur flux at the community level. In healthy oral microbiomes, sulfur is efficiently channeled into anabolic pathways—cysteine and methionine biosynthesis, heme synthesis—effectively sequestering it from volatile release. In the dysbiotic halitosis microbiome, the dominance of proteolytic *Prevotella* species generates a high flux of sulfur-containing amino acid catabolites that overwhelms a community with diminished biosynthetic capacity, resulting in net volatile sulfur compound accumulation and release. This sulfur assimilation failure model generates several specific, testable predictions: (i) metatranscriptomic analysis should reveal downregulation of *cysK*, *cysM*, and *metE/metH* gene expression in halitosis-associated communities relative to healthy controls; (ii) targeted metabolomic profiling should detect elevated free cysteine and methionine concentrations in halitosis oral fluid, reflecting reduced assimilation into protein-bound or biosynthetic pools; and (iii) supplementation with sulfur-assimilating commensals such as *H. parainfluenzae* should reduce volatile sulfur compound output in *in vitro* oral biofilm models without directly depleting proteolytic taxa. Notably, the concept of sulfur metabolic dysregulation as a driver of disease is not unique to the oral cavity. In the gut microbiome, impaired sulfur assimilation and the accumulation of hydrogen sulfide have been implicated in the pathogenesis of inflammatory bowel disease, where excess H_₂_S disrupts colonocyte energy metabolism and barrier integrity (19). Similarly, sulfate-reducing bacteria in the gut have been linked to disease activity through their role in diverting sulfur from host-beneficial pathways. Our findings suggest that an analogous systems-level principle may operate in the oral cavity: when the community’s capacity to assimilate sulfur into biosynthetic intermediates is diminished, toxic volatile sulfur compounds accumulate as metabolic overflow products. This cross-system parallel underscores the broader relevance of sulfur flux partitioning as a functional determinant of host-associated microbiome health (20).

Several limitations of this study merit consideration. First, functional predictions derived from PICRUSt2 are inferred from 16S rRNA gene amplicon data and therefore represent genomic potential rather than actual transcriptional activity or metabolite concentrations. The sulfur assimilation deficit we describe should accordingly be interpreted as a community-level genomic signature rather than a confirmed metabolic phenotype. Validation using metatranscriptomic, metaproteomic, or targeted metabolomic approaches—particularly direct quantification of intracellular cysteine and methionine pools—will be necessary to confirm whether the observed pathway suppression reflects genuine differences in enzyme expression and metabolic flux. Nonetheless, as a partial phenotype-level corroboration, we found that predicted sulfur pathway abundance was significantly correlated with directly measured volatile sulfur compound concentrations across all 90 participants (see Results), providing independent evidence that the predicted genomic signature is associated with the measured malodor phenotype even in the absence of direct metabolite quantification. Second, 16S rRNA V3–V4 amplicon sequencing has inherent limitations in species-level resolution due to the partial coverage of hypervariable regions and the reliance on reference database taxonomy. Third, oral gargle samples capture a pooled representation of the entire oral cavity rather than site-specific communities. Given that the tongue dorsum is widely regarded as the primary anatomical reservoir for volatile sulfur compound-producing bacteria (5), gargle-based sampling may dilute tongue-specific dysbiotic signals with microbiota from other oral niches, including buccal mucosa, hard palate, and saliva. Consequently, the Prevotella enrichment and other compositional signatures reported here should be interpreted as a community-level average across these niches rather than a direct readout of the tongue dorsum biofilm most proximally responsible for malodor production. While this approach offers practical advantages for clinical sampling and captures the integrated oral microbial community, future studies employing site-specific sampling of the tongue dorsum would provide higher resolution of the microbial communities most directly implicated in halitosis pathophysiology. Fourth, while halitosis status was defined using Twin Breasor II instrument-based volatile sulfur compound quantification, this device measures hydrogen sulfide and methyl mercaptan but does not capture the full spectrum of oral malodor compounds, including dimethyl sulfide. This is a relevant gap for our proposed sulfur assimilation failure model, as several of the suppressed pathways identified here—most notably the superpathway of sulfolactate degradation—lie mechanistically upstream of methanethiol/dimethyl sulfide-generating sulfur flux, such that undetected DMS production could not be directly incorporated into our VSC correlation analysis (see Results) and may represent an additional, unmeasured component of the malodor phenotype. Future studies employing more comprehensive volatile sulfur compound profiling alongside microbiome analysis would further strengthen mechanistic inference. Fifth, the significant age difference between groups (halitosis: mean 48.2 ± 11.2 years; normal: mean 27.8 ± 6.2 years; *P* < 0.001) represents an important limitation of the current study design. Age-related physiological changes—including reduced salivary flow, altered salivary pH and buffering capacity, hormonal shifts, cumulative periodontal and dental history, and a higher likelihood of undocumented medication use not captured by our exclusion criteria—could plausibly overlap with, and partially contribute to, the dysbiotic signals we attribute to halitosis. We addressed this concern using three converging analyses. First, halitosis status remained a significant independent predictor of community composition after age adjustment (PERMANOVA, *R*² = 0.030, *P* = 0.003). Second, a sensitivity analysis restricting PERMANOVA to age-overlapping subsets of the cohort confirmed that group separation persisted across all age-matched windows tested, including a stringent 22–45-year subset (*R*² = 0.054, *P* = 0.001) and an intentionally narrow 27–35-year subset with markedly reduced power (R² = 0.090, *P* = 0.018). Third, when the taxa identified as significant in the unadjusted genus- and species-level analyses were re-tested using an age-adjusted linear model, the direction of the halitosis-associated effect was preserved for every taxon (24/24 genera and 30/30 species), indicating no reversal of the reported associations. Formal statistical significance after age adjustment and multiple testing correction was retained for the majority of taxa (15/24 genera and 18/30 species remained significant at FDR < 0.05), while the remainder showed attenuated but directionally consistent associations, most likely reflecting reduced power from collinearity between age and halitosis status in this cohort rather than a true absence of effect. Several taxa central to the compositional signature described above, including *Peptostreptococcus stomatis*, *Veillonella rogosae*, and *Prevotella_7 melaninogenica* at the species level—retained formal significance after age adjustment, whereas *Prevotella_7* at the genus level and *Haemophilus parainfluenzae* showed attenuated but directionally consistent associations. Taken together, these analyses indicate that the direction of the reported taxonomic associations is not explained by age, and that the majority of individual taxon-level effects remain formally significant after adjustment; nonetheless, formal statistical confirmation of the full taxonomic signature independent of age will require age-matched cohorts with adequate power, and residual confounding at the level of individual taxa cannot be excluded given the observed attenuation for a subset of taxa. Future studies should employ age-matched recruitment to eliminate this source of ambiguity and to achieve full inferential power for taxon-level comparisons. It should also be noted that the LEfSe analysis used to highlight species-level biomarkers in the Abstract and Discussion (*P. melaninogenica*, *Peptostreptococcus stomatis*, *Haemophilus parainfluenzae*, and *Veillonella rogosae*) was not age-adjusted. Cross-referencing these LEfSe-identified taxa against the age-adjusted analysis above (Table S3), three of the four—*P*. *melaninogenica*, *Peptostreptococcus stomatis*, and *Veillonella rogosae*—remained among the species retaining formal significance (FDR < 0.05) after age adjustment; only *Haemophilus parainfluenzae* showed an attenuated but directionally consistent association. This largely consistent pattern strengthens confidence in the LEfSe-identified species as robust discriminant biomarkers, while formal taxon-level significance independent of age should still be confirmed in future age-matched cohorts. Sixth, this study was conducted in a single clinical center in South Korea, and the generalizability of these findings to other ethnic populations or geographic regions remains to be established. Additionally, this study did not include mock community positive controls or extraction negative controls, and no computational contaminant identification methods (e.g., decontam) were applied. Although oral gargle samples are expected to harbor high microbial biomass, reducing the risk of reagent-derived contamination, the absence of these controls limits our ability to formally assess potential contaminant sequences. Biological or technical replicates were not included in the sequencing design, precluding assessment of within-sample reproducibility. All library preparation and sequencing were performed in a single batch at Macrogen Inc., minimizing batch-effect confounding; however, the absence of formal blocking or randomization across batches should be noted. Furthermore, participants were recruited through convenience sampling at a single dental hospital, which may introduce selection bias that limits the representativeness of the study population. Finally, as an observational cross-sectional study, we cannot exclude the possibility that confounding factors—including diet, oral hygiene practices, and systemic health status—contribute to the observed microbiome differences. Collectively, these limitations point to clear priorities for future work: prospective recruitment of age-matched halitosis and control cohorts, site-specific sampling of the tongue dorsum biofilm, volatile sulfur compound quantification encompassing dimethyl sulfide alongside hydrogen sulfide and methyl mercaptan, and targeted metatranscriptomic or metabolomic validation of the sulfur assimilation failure model proposed here.

Notwithstanding these limitations, this study advances our understanding of halitosis pathophysiology in several important respects. The species-level resolution achieved through amplicon sequence variant-based analysis reveals clinically and functionally meaningful distinctions within the *Prevotella* genus that genus-level profiling would obscure. The identification of a predicted sulfur assimilation deficit as a systems-level functional hallmark of the halitosis microbiome provides a mechanistic hypothesis that is both testable and actionable: probiotic strategies aimed at restoring *H. parainfluenzae* or *V. rogosae* colonization, or prebiotic approaches designed to promote sulfur-assimilating metabolic activity, represent rational directions for future therapeutic development (21). Taken together, our findings establish a species-resolved and functionally grounded reference framework for halitosis-associated oral dysbiosis in East Asian adults and provide a foundation for targeted mechanistic validation and microbiome-based interventional studies.

## MATERIALS AND METHODS

### Study design and participant recruitment

This study was conducted using samples and associated clinical data distributed by the Apple Tree Oral Biobank (Appletree Dental Hospital, Goyang-si, Gyeonggi-do, Republic of Korea), a biobank registered under the National Biobank of Korea (Korea Disease Control and Prevention Agency, Project No. 2024ER050701). Samples had been collected from a total of 90 adult donors between May and December 2024, comprising 45 individuals with halitosis (halitosis group) and 45 healthy controls (normal group). Donors were eligible if they were aged 19 years or older and had provided written informed consent for biobank registration and subsequent research use. Individuals with a diagnosis of periodontal disease, systemic illness, or a history of antibiotic use within the preceding 3 months were excluded, as were current smokers and individuals who had used probiotic supplements within 1 month prior to sample collection. Halitosis was defined as an instrument-based halitosis score of ≥ 1 on the Twin Breasor II diagnostic scale (iSenLab Inc., Seongnam, Republic of Korea), corresponding to a volatile sulfur compound measurement of hydrogen sulfide ≥112 ppb or methyl mercaptan ≥26 ppb. Healthy controls were defined as individuals with no subjective or objective evidence of oral malodor (Twin Breasor II score = 0; hydrogen sulfide <112 ppb and methyl mercaptan <26 ppb). The Twin Breasor II is a certified medical-grade exhaled breath analyzer capable of simultaneously measuring hydrogen sulfide and methyl mercaptan concentrations in oral air in real time, and represents the only domestically developed and clinically validated instrument in Korea enabling simultaneous quantification of both major volatile sulfur compounds associated with halitosis.

### Sample collection

Oral gargle samples were obtained from the Apple Tree Oral Biobank as described above. All samples had been collected from consenting donors during a single clinical visit in the morning, following an overnight fast of at least 8 h. Participants were instructed to refrain from tooth brushing prior to sample collection to preserve the native oral microbial community. Briefly, participants gargled with 10 mL of a commercially available alcohol-free oral rinse solution (CoolSense Stick Gargle; Docsmedi, Republic of Korea) for 30 s, after which the gargling fluid was collected in a sterile tube and immediately placed on ice. All samples were stored at −80°C until DNA extraction.

### DNA extraction and 16S rRNA gene library preparation

Genomic DNA was extracted from all samples using the LaboPass Bacteria Genomic DNA Isolation Kit (Cosmo Genetech, Seoul, Republic of Korea) following the manufacturer’s protocol. The V3–V4 hypervariable region of the 16S rRNA gene was amplified using the universal primer pair 341F (5′-CCTACGGGNGGCWGCAG-3′) and 805R (5′-GACTACHVGGGTATCTAATCC-3′). Library preparation was performed using the Metagenome Amplicon Library kit, and paired-end sequencing (2 × 301 bp) was carried out on the Illumina MiSeq platform at Macrogen Inc. (Seoul, Republic of Korea).

### Sequence processing and amplicon sequence variant inference

Raw sequencing reads were processed using the DADA2 package (v1.28) (22) in R (v4.3.3). Adapter and primer sequences were removed prior to quality filtering. Reads were quality-filtered and trimmed using the *filterAndTrim* function with the following parameters: forward reads truncated to 250 bp, reverse reads truncated to 200 bp, maximum expected errors of 2 for both forward and reverse reads, and truncQ = 2. Samples with fewer than 10,000 reads after quality filtering were excluded from downstream analysis. Error rates were estimated using the *learnErrors* function across all samples jointly. Denoising was performed using the *dada* function with pseudo-pooling (pool = “pseudo”) to improve sensitivity for low-abundance variants. Forward and reverse reads were merged using *mergePairs*, and sequence variants shorter than 350 bp were removed. Chimeric sequences were identified and removed using the *removeBimeraDenovo* function with the consensus method. Taxonomic classification was performed against the SILVA reference database (v138.1) using *assignTaxonomy* with a minimum bootstrap confidence of 50%, followed by species-level assignment using *addSpecies*. The genus designation *“Prevotella*_7” follows the SILVA v138.1 taxonomy and is retained in all DADA2/SILVA-based analyses reported here, including genus- and species-level differential abundance, core microbiome, age-adjusted, and power analyses, to maintain consistency with the reference database classification. For readability, narrative descriptions in the Abstract and Discussion, as well as the independently processed LEfSe analysis (performed via the Galaxy platform), refer to this taxon as *P. melaninogenica*, consistent with common usage in the halitosis literature; the two designations refer to the same organism.

### Diversity analysis and community-level comparisons

All diversity analyses were performed in R using the vegan (v2.7.2) (23) and phyloseq (v1.46.0) (24) packages. Prior to alpha and beta diversity calculations, samples were rarefied to the minimum sequencing depth across all samples (22,209 reads; rrarefy function, vegan package, seed = 42) to control for uneven sequencing depth; differential abundance analyses were performed on non-rarefied relative abundances (see below). For alpha diversity, observed species richness, Shannon index, Simpson index, and Faith’s phylogenetic diversity were calculated per sample. A phylogenetic tree was constructed from aligned ASV sequences using MAFFT (v7.525) (25) and FastTree (v2.2.0) (26). Statistical comparisons of alpha diversity indices between groups were performed using the Wilcoxon rank-sum test with Benjamini–Hochberg false discovery rate correction. Beta diversity was assessed using Bray–Curtis dissimilarity, visualized by PCoA, and tested for group-level separation using PERMANOVA (*adonis2* function, 999 permutations). Homogeneity of multivariate dispersions was assessed using the *betadisper* function followed by a permutation test (999 permutations). The Firmicutes/Bacteroidota ratio was calculated from phylum-level relative abundances and compared between groups using the Wilcoxon rank-sum test. To account for the significant age difference between groups (Table 1), PERMANOVA was additionally performed with age included as a covariate in the model formula (community dissimilarity ~ age + group, 999 permutations), and the independent explanatory power of group membership was assessed after adjusting for age. Sex distribution did not differ significantly between the halitosis and healthy control groups (*P* = 0.582, chi-squared test; Table 1) and was therefore not included as a covariate in the PERMANOVA or differential abundance models.

### Differential abundance analysis

Relative abundances were calculated by dividing per-sample ASV counts by the total read count per sample. ASVs were collapsed to genus and species levels by summing relative abundances of ASVs sharing the same taxonomic assignment. For species-level analysis, ASVs were assigned a label comprising genus and species names; ASVs classified only to genus level were labeled as [Genus] sp. Prior to differential abundance testing, genera and species were required to be detected (nonzero relative abundance) in at least 9 of 90 samples (≥10% prevalence) to reduce the multiple-testing burden associated with extremely rare taxa. Differentially abundant taxa were identified using the Wilcoxon rank-sum test with Benjamini–Hochberg FDR correction, applied independently at genus and species levels among taxa passing this prevalence filter. Taxa with FDR < 0.05 were considered statistically significant. Core microbiome analysis was performed by identifying species present in at least 70% of samples within each group. As a sensitivity analysis to further address the age difference between groups, genus- and species-level relative abundances were arcsine-square-root transformed and modeled using linear regression with halitosis status and age as covariates (transformed abundance ~ group + age) in R (v4.3.3). The *P* value associated with the group coefficient was extracted for each taxon and corrected for multiple testing using the Benjamini–Hochberg procedure (FDR < 0.05 considered significant); the direction of the age-adjusted group effect was compared with that of the unadjusted Wilcoxon rank-sum test. This age-adjusted re-analysis was restricted to the specific set of taxa already identified as significant in the primary unadjusted screen, rather than re-screening all tested taxa, in order to evaluate the robustness of these pre-specified, hypothesis-driven findings to age adjustment without incurring the additional multiple-testing burden associated with a full exploratory re-scan; this sensitivity analysis design follows common practice for confounder-adjustment checks of previously identified biomarkers.

### LEfSe analysis

Linear discriminant analysis effect size (LEfSe) (27) was used to identify species-level biomarkers discriminating the halitosis and healthy control groups. LEfSe was run using the Galaxy web-based platform with default parameters (α = 0.05 for the Kruskal–Wallis test; LDA score threshold = 2.0). Only species with an LDA score greater than 2.0 were considered as discriminant biomarkers.

### Functional pathway prediction

Functional pathway abundances were predicted using PICRUSt2 (v2.5.2) (28) with default parameters. Briefly, ASV sequences were placed into a reference phylogenetic tree, and functional gene content was predicted using the hidden-state prediction approach. Prediction reliability was assessed using the weighted NSTI, which quantifies the phylogenetic distance between each ASV and its nearest reference genome in the PICRUSt2 tree; ASVs exceeding the default NSTI cutoff of 2.0 were excluded from functional prediction (14 of 4,598 ASVs excluded). Sample-level weighted NSTI (community-weighted mean across retained ASVs) was compared between groups using the Wilcoxon rank-sum test. MetaCyc pathway abundances were predicted from the inferred gene family abundances using the MinPath algorithm. Pathway abundances were normalized to relative abundance per sample. Differential pathway analysis between groups was performed using the Wilcoxon rank-sum test with Benjamini–Hochberg FDR correction in Python using the SciPy (v1.16.3) and statsmodels (v0.14.6) packages. Sulfur metabolism-related pathways were identified by keyword matching against MetaCyc pathway descriptions, including terms related to sulfur, sulfate, sulfide, sulfo-, sulfhydryl, thiosulfate, thiol, mercaptan, methanethiol, hydrogen sulfide, cysteine, methionine, and heme biosynthesis, yielding 27 candidate pathways. Benjamini–Hochberg FDR correction for this analysis was applied independently within this pre-specified set of 27 sulfur-related pathways, separately from the genome-wide correction applied to the full pathway set (Fig. 6A). Fig. 6B displays 14 of these 27 pathways that reached nominal significance (*P* < 0.05).

### Correlation with directly measured volatile sulfur compounds

To evaluate whether predicted sulfur pathway suppression corresponds to the directly measured malodor phenotype, a composite sulfur pathway score was constructed for each sample as the sum of relative abundances of eight core MetaCyc pathways, selected from the 14 sulfur metabolism-related pathways shown in Fig. 6B based on direct annotation to sulfate assimilation, cysteine/methionine biosynthesis, or heme biosynthesis (Table S5). This composite score, together with each of the eight individual pathways, was correlated with directly measured hydrogen sulfide and methyl mercaptan concentrations (Twin Breasor II) across all 90 participants using Spearman’s rank correlation. To assess whether these associations were independent of the age difference between groups, partial Spearman correlations controlling for age were additionally computed using the *partial_corr* function in the pingouin package (v0.6.1) for Python (Table S5).

### Statistical analysis

All statistical analyses were performed in R (v4.3.3) and Python (v3.9.23). Group comparisons were conducted using the Wilcoxon rank-sum test. Multiple testing correction was applied using the Benjamini–Hochberg procedure throughout; corrected *P* values are reported as FDR or q-values. Statistical significance was defined as FDR < 0.05 unless otherwise stated. Data visualization was performed using ggplot2 (v3.4.4) (29) in R and matplotlib (v3.10.6) in Python.

### Post-hoc power analysis

Because closed-form power formulas do not exist for permutation-based multivariate tests such as PERMANOVA, statistical power was estimated empirically via subsampling. For community-level power, subsets of n individuals per group (*n* = 4–45) were randomly drawn without replacement from the full halitosis and normal groups (200–300 iterations per sample size), and PERMANOVA (999 permutations) was performed on the Bray–Curtis distance matrix restricted to each subsample; power at each sample size was defined as the proportion of iterations yielding *P* < 0.05. An analogous subsampling procedure was used to estimate power for representative species-level taxa, substituting the Wilcoxon rank-sum test for PERMANOVA. All power analyses were implemented in Python (v3.9.23) using scikit-bio (v0.7.3) and SciPy (v1.16.3).

## Data Availability

The raw sequencing data generated in this study have been deposited in the NCBI Sequence Read Archive under BioProject accession number PRJNA1427141 (submission ID: SUB16018861; BioSample accessions: SAMN55904799–SAMN55904888) 30. The completed STORMS checklist for this study is available at https://doi.org/10.5281/zenodo.18824829.

## SUPPLEMENTAL MATERIAL

Supplemental figures spectrum.00906-26-s0001.pdf Fig. S1 to S11.

Supplemental tables spectrum.00906-26-s0002.docx Tables S1 to S6.

ASM does not own the copyrights to Supplemental Material that may be linked to, or accessed through, an article. The authors have granted ASM a non-exclusive, world-wide license to publish the Supplemental Material files. Please contact the corresponding author directly for reuse.
