# Supplementary material for: Prevotella-dominated dysbiosis and predicted suppression of sulfur biosynthesis pathways associated with halitosis in Korean adults
Source: Microbiol Spectr. 2026 Sep 16;14(10):e00906-26. doi: 10.1128/spectrum.00906-26 (PMC13689370; doi:10.1128/spectrum.00906-26)
Supplement: Supplementary file 1 [file spectrum.00906-26-s0001.pdf]

Fig. S1

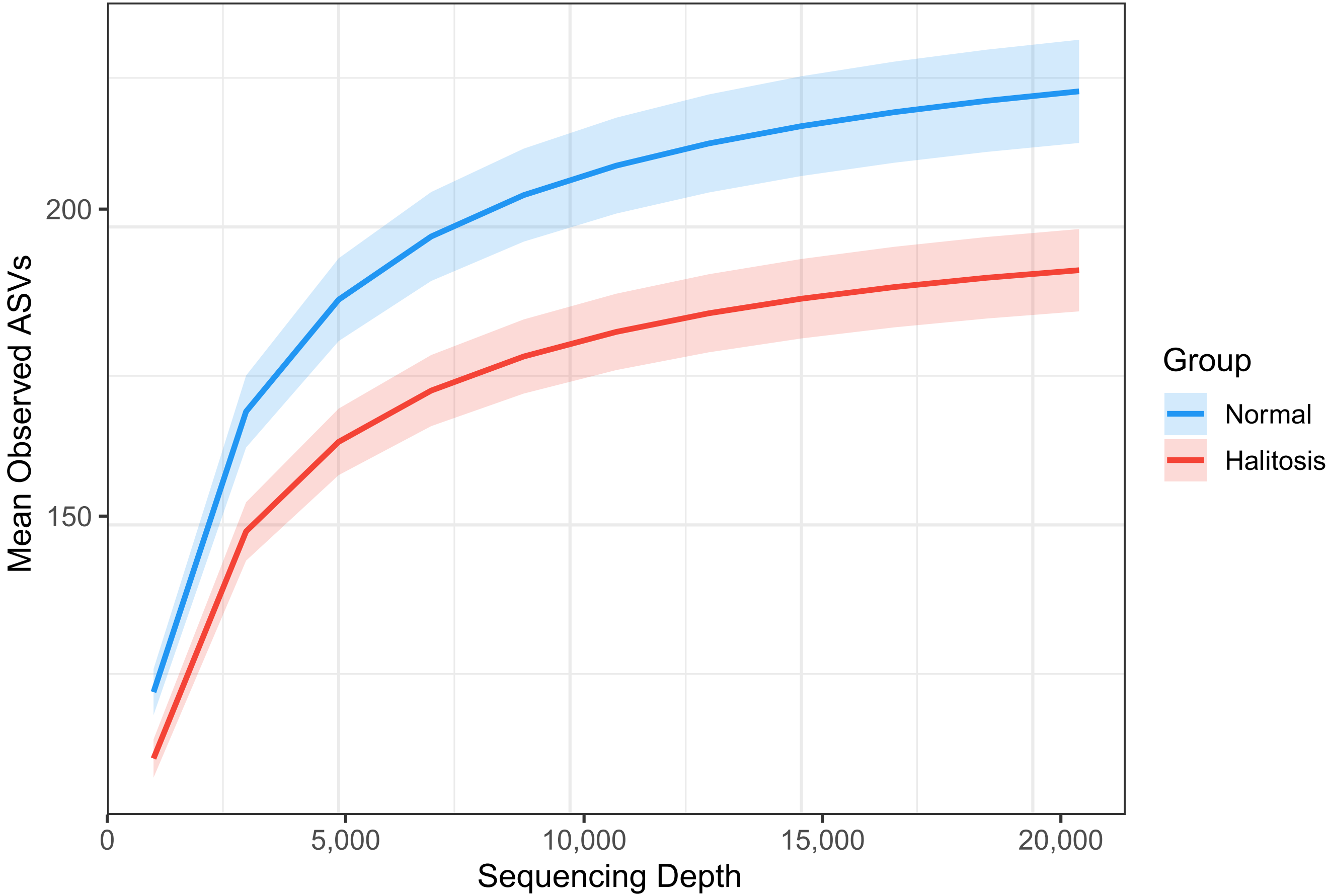

Mean Observed ASVs
200
150
0
5,000
10,000
15,000
20,000
Sequencing Depth
Group
Normal
Halitosis

Fig. S2

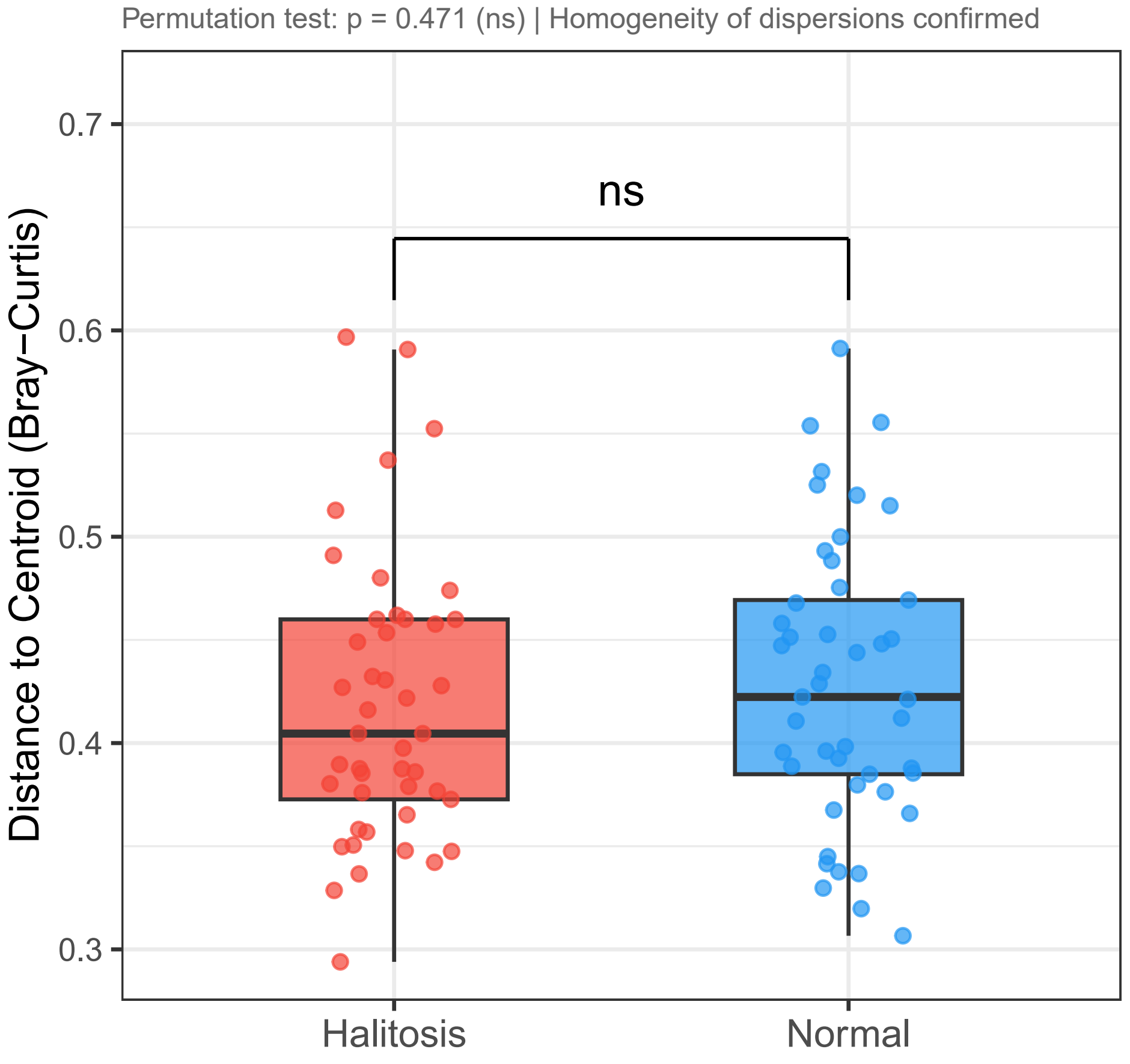
Permutation test: p = 0.471 (ns) | Homogeneity of dispersions confirmed
ns
Distance to Centroid (Bray−Curtis)
0.7
0.6
0.5
0.4
0.3
Halitosis
Normal

Fig. S3

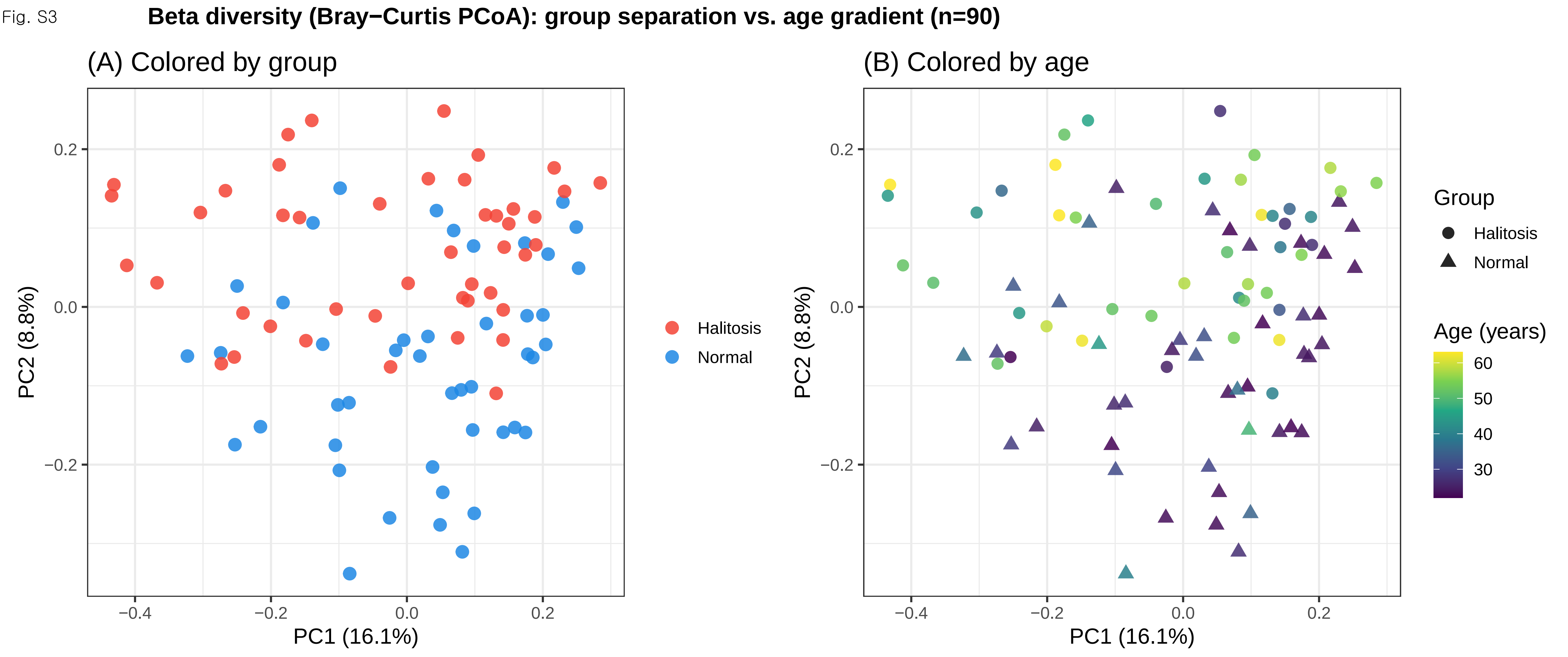
Beta diversity (Bray−Curtis PCoA): group separation vs. age gradient (n=90)
(A) Colored by group
(B) Colored by age
PC2 (8.8%)
PC1 (16.1%)
0.2
0.0
−0.2
−0.4
Halitosis
Normal
Group
Age (years)
60
50
40
30

Fig. S4

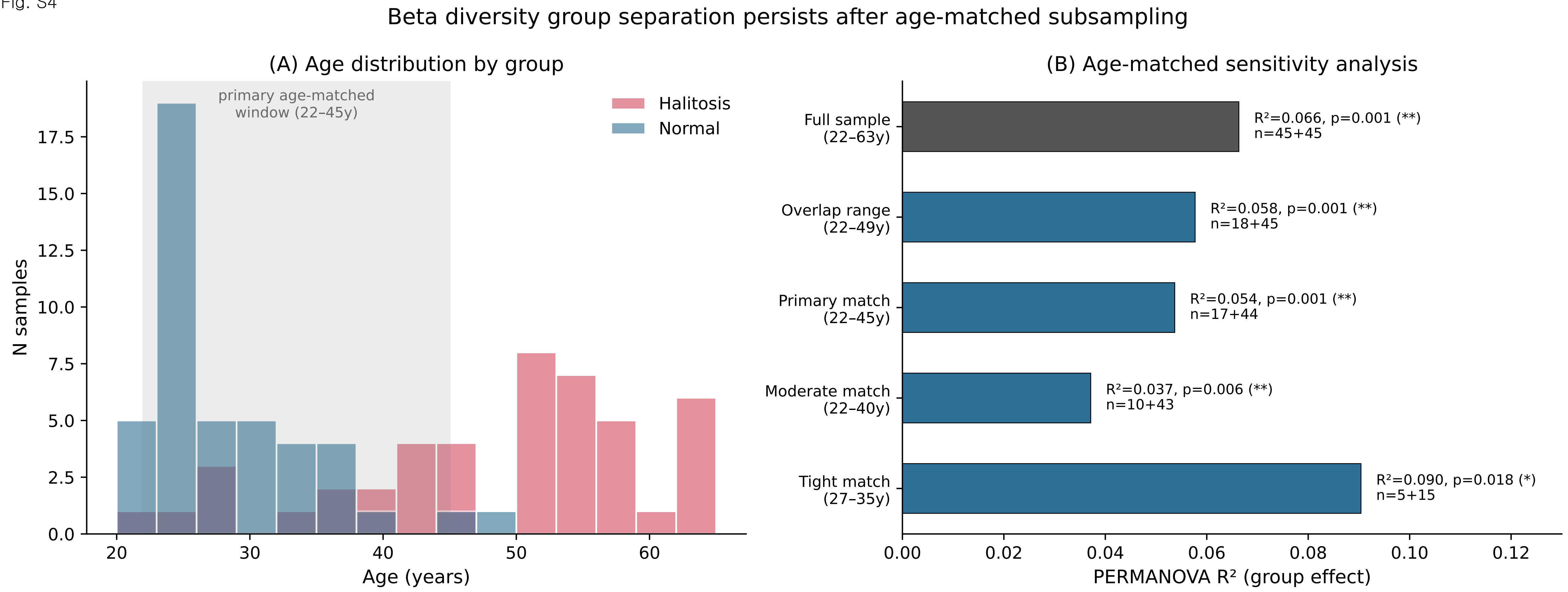

Beta diversity group separation persists after age-matched subsampling
(A) Age distribution by group
primary age-matched
window (22–45y)
Halitosis
Normal
N samples
Age (years)
(B) Age-matched sensitivity analysis
Full sample
(22–63y)
R²=0.066, p=0.001 (**)
n=45+45
Overlap range
(22–49y)
R²=0.058, p=0.001 (**)
n=18+45
Primary match
(22–45y)
R²=0.054, p=0.001 (**)
n=17+44
Moderate match
(22–40y)
R²=0.037, p=0.006 (**)
n=10+43
Tight match
(27–35y)
R²=0.090, p=0.018 (*)
n=5+15
PERMANOVA R² (group effect)

Fig. S5

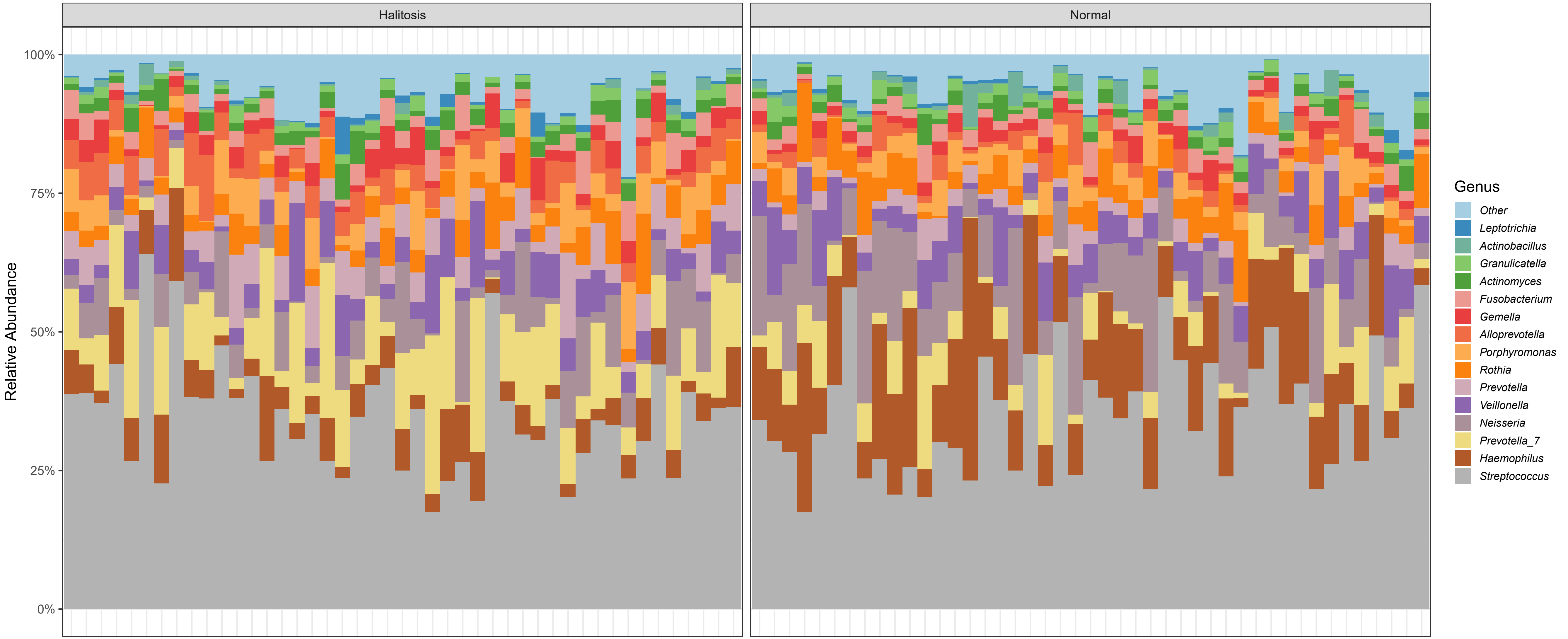

Halitosis
Normal
Relative Abundance
100%
75%
50%
25%
0%
Genus
Other
Leptotrichia
Actinobacillus
Granulicatella
Actinomyces
Fusobacterium
Gemella
Alloprevotella
Porphyromonas
Rothia
Prevotella
Veillonella
Neisseria
Prevotella_7
Haemophilus
Streptococcus

Fig. S6

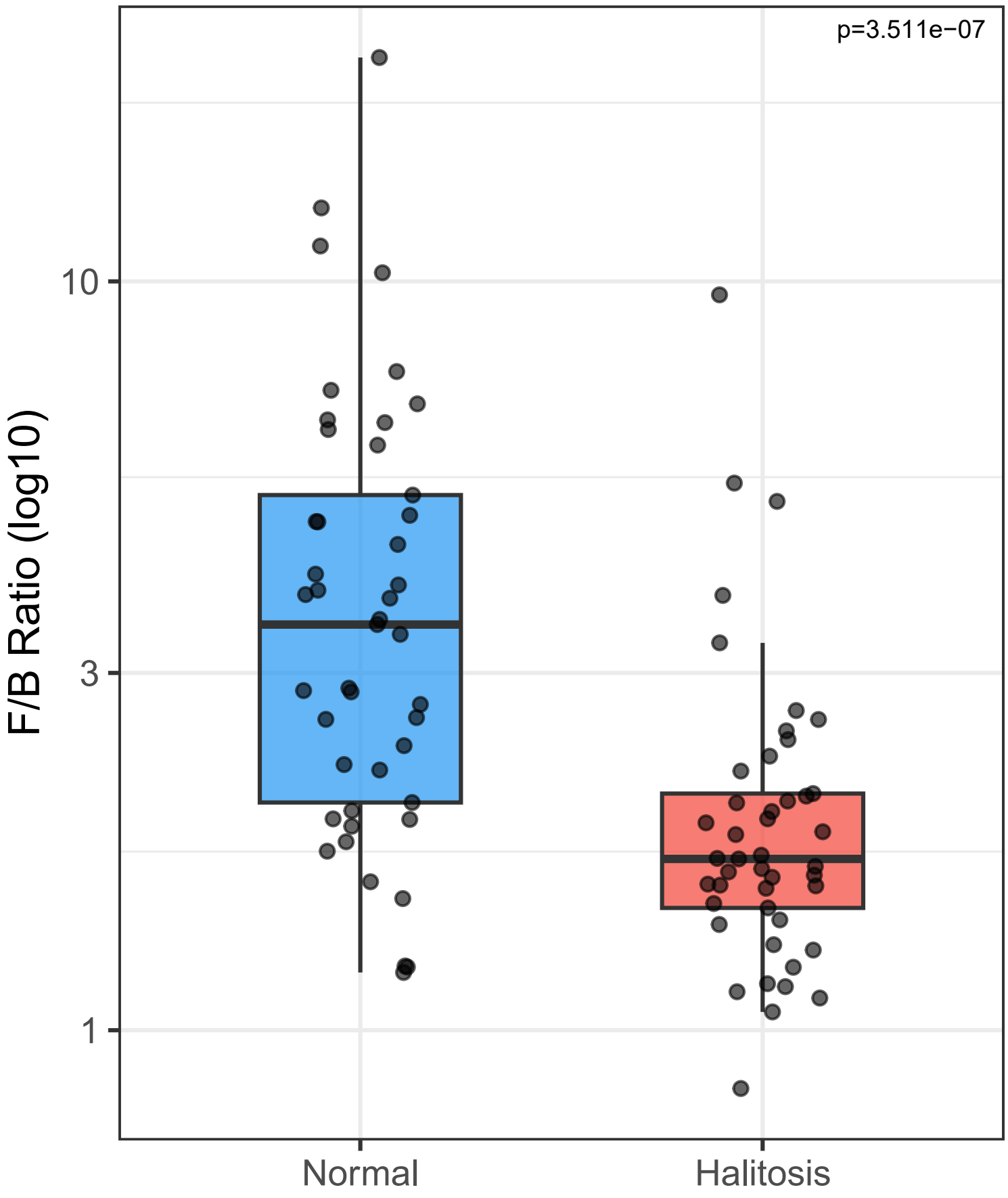

Fig. S7

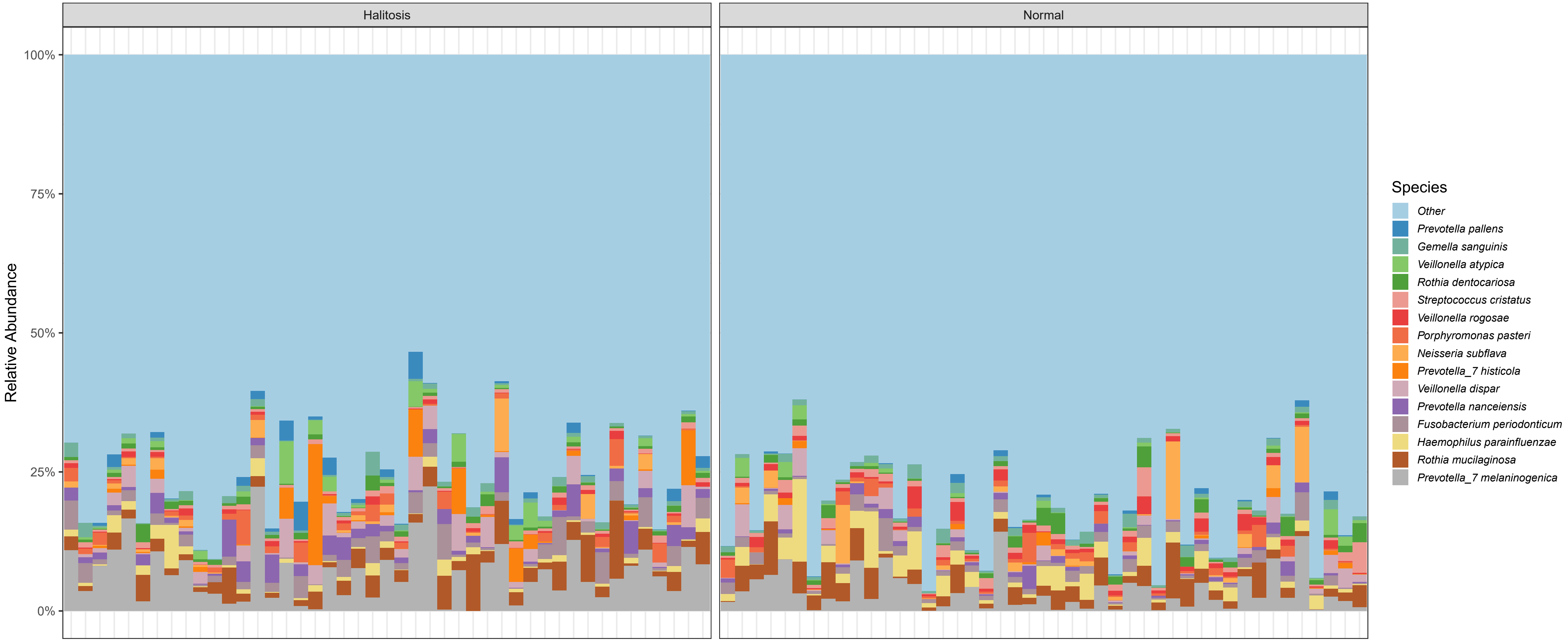
Halitosis
Normal
100%
75%
50%
25%
0%
Relative Abundance
Species
Other
Prevotella pallens
Gemella sanguinis
Veillonella atypica
Rothia dentocariosa
Streptococcus cristatus
Veillonella rogosae
Porphyromonas pasteri
Neisseria subflava
Prevotella_7 histicola
Veillonella dispar
Prevotella nanceiensis
Fusobacterium periodonticum
Haemophilus parainfluenzae
Rothia mucilaginosa
Prevotella_7 melaninogenica

Fig. S8

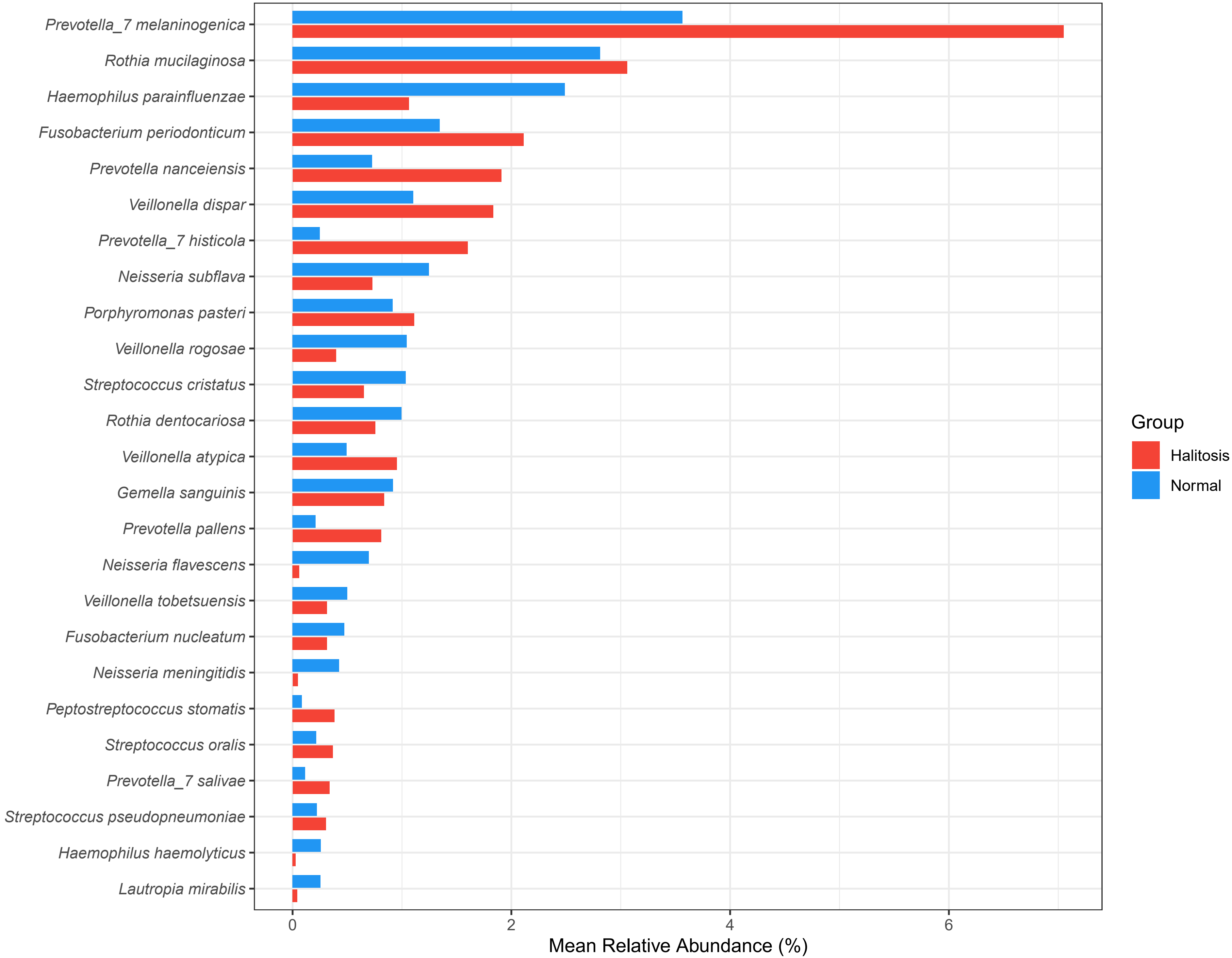
Prevotella_7 melaninogenica
Rothia mucilaginosa
Haemophilus parainfluenzae
Fusobacterium periodonticum
Prevotella nanceiensis
Veillonella dispar
Prevotella_7 histicola
Neisseria subflava
Porphyromonas pasteri
Veillonella rogosae
Streptococcus cristatus
Rothia dentocariosa
Veillonella atypica
Gemella sanguinis
Prevotella pallens
Neisseria flavescens
Veillonella tobetsuensis
Fusobacterium nucleatum
Neisseria meningitidis
Peptostreptococcus stomatis
Streptococcus oralis
Prevotella_7 salivae
Streptococcus pseudopneumoniae
Haemophilus haemolyticus
Lautropia mirabilis
0
2
4
6
Mean Relative Abundance (%)
Group
Halitosis
Normal

Fig. S9

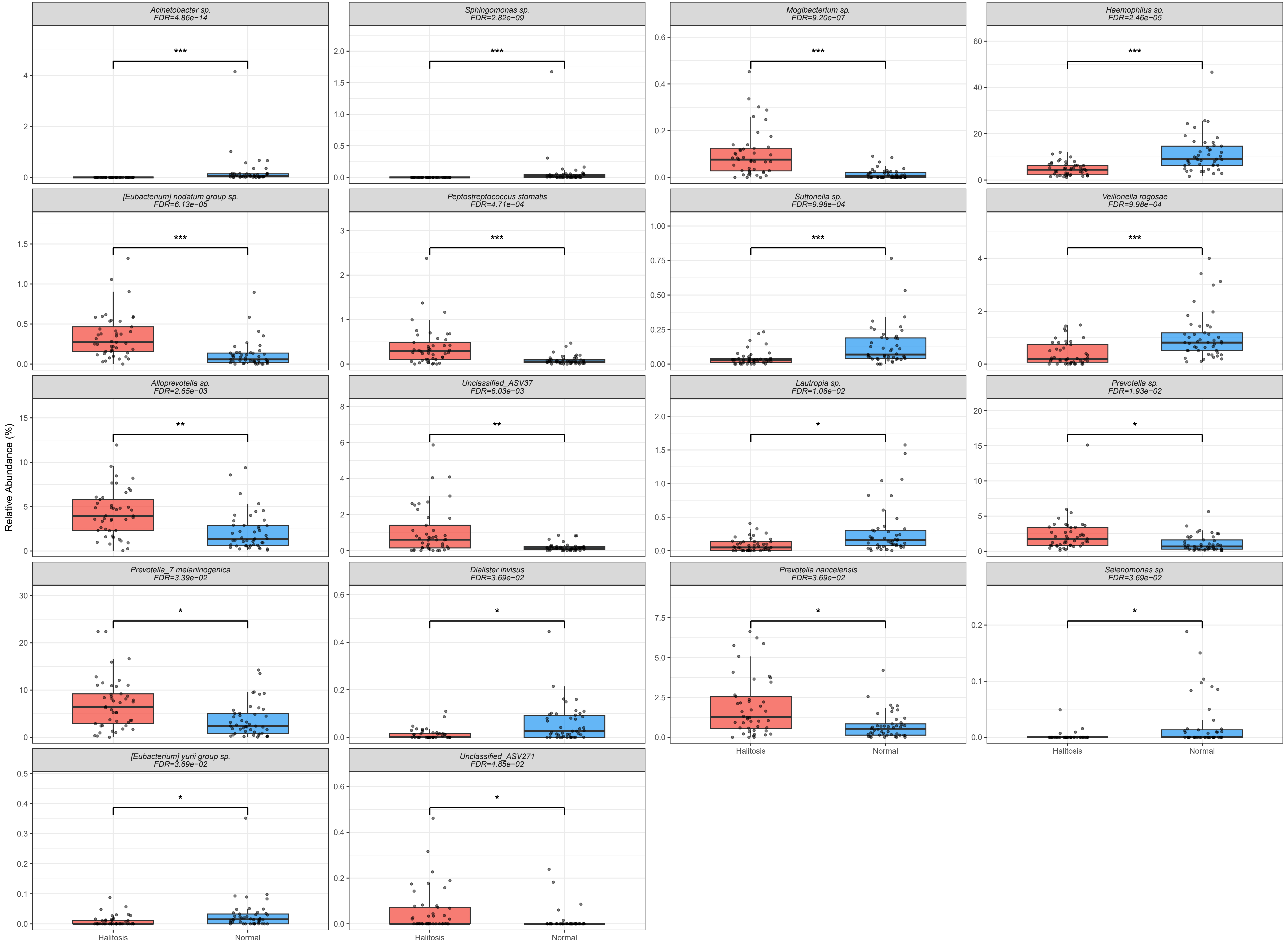

Acinetobacter sp.
FDR=4.86e−14
Sphingomonas sp.
FDR=2.82e−09
Mogibacterium sp.
FDR=9.20e−07
Haemophilus sp.
FDR=2.46e−05
[Eubacterium] nodatum group sp.
FDR=6.13e−05
Peptostreptococcus stomatis
FDR=4.71e−04
Suttonella sp.
FDR=9.98e−04
Veillonella rogosae
FDR=9.98e−04
Alloprevotella sp.
FDR=2.65e−03
Unclassified_ASV37
FDR=6.03e−03
Lautropia sp.
FDR=1.08e−02
Prevotella sp.
FDR=1.93e−02
Prevotella_7 melaninogenica
FDR=3.39e−02
Dialister invisus
FDR=3.69e−02
Prevotella nanceiensis
FDR=3.69e−02
Selenomonas sp.
FDR=3.69e−02
[Eubacterium] yurii group sp.
FDR=3.69e−02
Unclassified_ASV271
FDR=4.85e−02
Relative Abundance (%)
Halitosis
Normal
***
**
*

Fig. S10

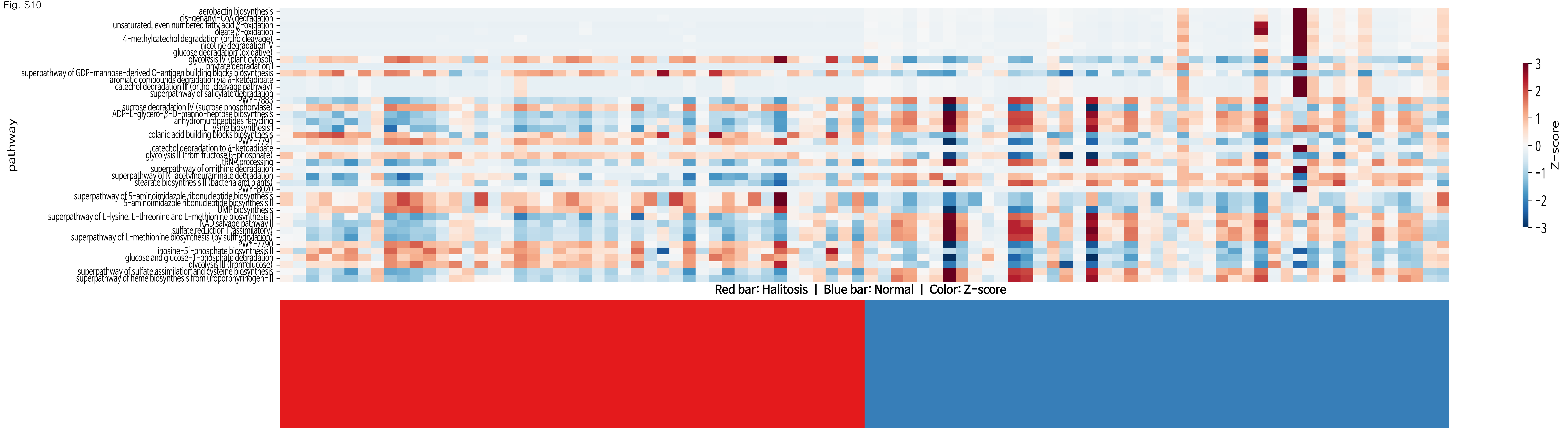
pathway
aerobactin biosynthesis
cis-genanyl-CoA degradation
unsaturated, even numbered fatty acid β-oxidation
oleate β-oxidation
4-methylcatechol degradation (ortho cleavage)
nicotine degradation IV
glucose degradation (oxidative)
glycolysis IV (plant cytosol)
phytate degradation I
superpathway of GDP-mannose-derived O-antigen building blocks biosynthesis
aromatic compounds degradation via β-ketoadipate
catechol degradation III (ortho-cleavage pathway)
superpathway of salicylate degradation
PWY-7883
sucrose degradation IV (sucrose phosphorylase)
ADP-L-glycero-β-D-manno-heptose biosynthesis
anhydromuropeptides recycling
L-lysine biosynthesis I
colanic acid building blocks biosynthesis
PWY-7791
catechol degradation to β-ketoadipate
glycolysis II (from fructose 6-phosphate)
tRNA processing
superpathway of ornithine degradation
superpathway of N-acetylneuraminate degradation
stearate biosynthesis II (bacteria and plants)
PWY-8020
superpathway of 5-aminoimidazole ribonucleotide biosynthesis
5-aminoimidazole ribonucleotide biosynthesis II
UMP biosynthesis
superpathway of L-lysine, L-threonine and L-methionine biosynthesis II
NAD salvage pathway II
sulfate reduction I (assimilatory)
superpathway of L-methionine biosynthesis (by sulfhydrylation)
PWY-7790
inosine-5'-phosphate biosynthesis II
glucose and glucose-1-phosphate degradation
glycolysis III (from glucose)
superpathway of sulfate assimilation and cysteine biosynthesis
superpathway of heme biosynthesis from uroporphyrinogen-III
Red bar: Halitosis | Blue bar: Normal | Color: Z-score
3
2
1
0
−1
−2
−3
Z-score

Fig. S11

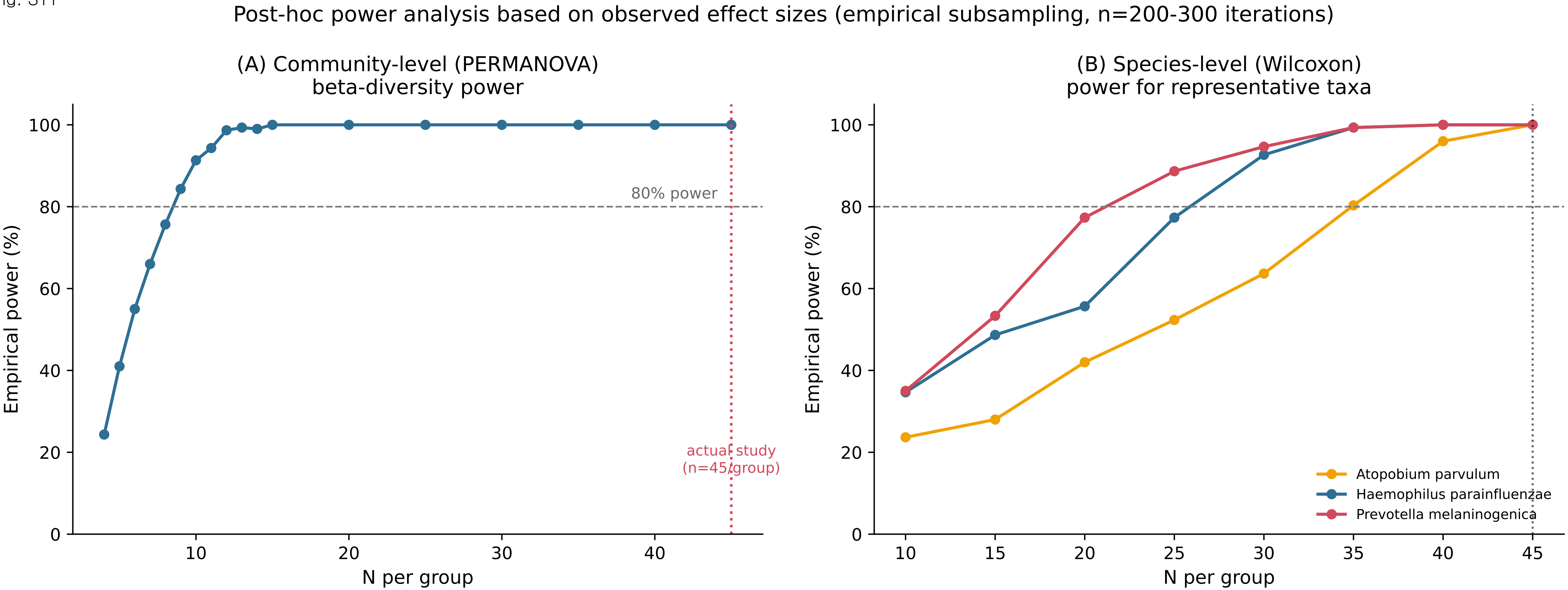

Post-hoc power analysis based on observed effect sizes (empirical subsampling, n=200-300 iterations)
(A) Community-level (PERMANOVA)
beta-diversity power
Empirical power (%)
N per group
80% power
actual study
(n=45/group)
(B) Species-level (Wilcoxon)
power for representative taxa
Empirical power (%)
N per group
Atopobium parvulum
Haemophilus parainfluenzae
Prevotella melaninogenica
