# Supplementary material for: Prevotella-dominated dysbiosis and predicted suppression of sulfur biosynthesis pathways associated with halitosis in Korean adults
Source: Microbiol Spectr. 2026 Sep 16;14(10):e00906-26. doi: 10.1128/spectrum.00906-26 (PMC13689370; doi:10.1128/spectrum.00906-26)
Supplement: Supplementary file 2 [file spectrum.00906-26-s0002.docx]

**Table S1. Age-adjusted PERMANOVA results for Bray–Curtis beta diversity.**

PERMANOVA was performed using the adonis2 function (vegan package, 999 permutations) with Bray–Curtis dissimilarity. Sequential sums of squares (by = "terms") were used in Models 2 and 3 to assess the independent contribution of each variable after accounting for the other.

Model 1: Group only

Formula: bc_dist ~ Group

| Term | df | SumOfSqs | R² | F | p-value |
| --- | --- | --- | --- | --- | --- |
| Group | 1 | 0.9039 | 0.0520 | 4.826 | 0.001 *** |
| Residual | 88 | 16.4817 | 0.9480 |  |  |
| Total | **89** | **17.3856** | **1.0000** |  |  |

Model 2: Age + Group (age-adjusted; sequential SS)

Formula: bc_dist ~ Age + Group

| Term | df | SumOfSqs | R² | F | p-value |
| --- | --- | --- | --- | --- | --- |
| Age | 1 | 0.7186 | 0.0413 | 3.873 | 0.001 *** |
| Group | 1 | 0.5246 | 0.0302 | 2.827 | **0.003 **** |
| Residual | 87 | 16.1424 | 0.9285 |  |  |
| Total | **89** | **17.3856** | **1.0000** |  |  |

Model 3: Group + Age (group-adjusted; sequential SS)

Formula: bc_dist ~ Group + Age

| Term | df | SumOfSqs | R² | F | p-value |
| --- | --- | --- | --- | --- | --- |
| Group | 1 | 0.9039 | 0.0520 | 4.872 | 0.001 *** |
| Age | 1 | 0.3393 | 0.0195 | 1.828 | 0.024 * |
| Residual | 87 | 16.1424 | 0.9285 |  |  |
| Total | **89** | **17.3856** | **1.0000** |  |  |

*, p < 0.05; **, p < 0.01; ***, p < 0.001.

R², proportion of variance explained. F, pseudo-F statistic.

The significance of Group in Model 2 (R² = 0.030, p = 0.003) confirms that halitosis group membership is an independent predictor of oral microbial community composition after controlling for age.

**Table S2. AgeMatch. Age-matched sensitivity analysis of beta-diversity group separation.**

PERMANOVA (Bray-Curtis dissimilarity, 999 permutations) was repeated on age-overlapping subsets of the cohort to test whether halitosis-associated community separation persisted independent of the age difference between groups. Group separation remained statistically significant across every age-matched window tested.

| **Subset** | **Age range (y)** | **N total** | **N halitosis** | **N normal** | **Pseudo-F** | **R²** | **p-value** | **Permutations** |
| --- | --- | --- | --- | --- | --- | --- | --- | --- |
| Full sample (all ages) | 22–63 | 90 | 45 | 45 | 6.252 | 0.066 | 0.001 | 999 |
| Full normal-range overlap | 22–49 | 63 | 18 | 45 | 3.736 | 0.058 | 0.001 | 999 |
| Primary age-matched subset | 22–45 | 61 | 17 | 44 | 3.347 | 0.054 | 0.001 | 999 |
| Moderate match | 22–40 | 53 | 10 | 43 | 1.966 | 0.037 | 0.006 | 999 |
| Tight match (underpowered) | 27–35 | 20 | 5 | 15 | 1.789 | 0.090 | 0.018 | 999 |

**Table S3. Age-adjusted differential abundance of genus- and species-level taxa significant in the unadjusted Wilcoxon analysis.**

Taxa shown were significant at FDR < 0.05 in the primary (unadjusted) Wilcoxon rank-sum comparison at the genus (n = 24) or species (n = 30) level. Each taxon was re-tested using an age-adjusted linear model (arcsine-square-root-transformed relative abundance ~ group + age); the group-coefficient p-value was extracted and corrected for multiple testing using the Benjamini–Hochberg procedure, applied within this targeted set of 24 genera and, separately, 30 species. The direction of the halitosis-associated effect (“Direction (uncorrected)”) was preserved for 100% of taxa (24/24 genera, 30/30 species) after age adjustment. Formal statistical significance at FDR < 0.05 after age adjustment was retained for 15 of 24 genera and 18 of 30 species (“Sig. after age adj.” column); attenuation for the remaining taxa most likely reflects reduced statistical power arising from collinearity between age and halitosis status in this cohort, rather than a reversal or absence of the underlying association.

| **Taxon** | **Level** | **Direction (uncorrected)** | **Wilcoxon FDR** | **Age-adj. FDR** | **Sig. after age adj.** |
| --- | --- | --- | --- | --- | --- |
| *[Acinetobacter] sp.* | Species | Higher in Normal | 5.39e-15 | 0.003 | Yes |
| *[Sphingomonas] sp.* | Species | Higher in Normal | 3.12e-10 | 0.017 | Yes |
| *[Mogibacterium] sp.* | Species | Higher in Halitosis | 1.02e-7 | 0.007 | Yes |
| *[Haemophilus] sp.* | Species | Higher in Normal | 2.73e-6 | 0.036 | Yes |
| *[[Eubacterium] nodatum group] sp.* | Species | Higher in Halitosis | 6.79e-6 | 0.04 | Yes |
| *Peptostreptococcus stomatis* | Species | Higher in Halitosis | 5.22e-5 | 0.003 | Yes |
| *Veillonella rogosae* | Species | Higher in Normal | 1.11e-4 | 0.039 | Yes |
| *[Suttonella] sp.* | Species | Higher in Normal | 1.11e-4 | 0.012 | Yes |
| *[Alloprevotella] sp.* | Species | Higher in Halitosis | 2.94e-4 | 0.003 | Yes |
| *[Lautropia] sp.* | Species | Higher in Normal | 1.32e-3 | 0.012 | Yes |
| *[Prevotella] sp.* | Species | Higher in Halitosis | 2.34e-3 | 0.387 | No |
| *Prevotella_7 melaninogenica* | Species | Higher in Halitosis | 4.07e-3 | 0.042 | Yes |
| *Dialister invisus* | Species | Higher in Normal | 4.34e-3 | 0.036 | Yes |
| *Prevotella nanceiensis* | Species | Higher in Halitosis | 4.34e-3 | 3.49e-5 | Yes |
| *[Selenomonas] sp.* | Species | Higher in Normal | 4.34e-3 | 0.027 | Yes |
| *[[Eubacterium] yurii group] sp.* | Species | Higher in Normal | 4.34e-3 | 0.068 | No |
| *Selenomonas noxia* | Species | Higher in Normal | 6.82e-3 | 0.019 | Yes |
| *Haemophilus parainfluenzae* | Species | Higher in Normal | 7.75e-3 | 0.226 | No |
| *[Veillonella] sp.* | Species | Higher in Normal | 9.32e-3 | 0.017 | Yes |
| *[Actinobacillus] sp.* | Species | Higher in Normal | 9.34e-3 | 0.152 | No |
| *Johnsonella ignava* | Species | Higher in Normal | 1.94e-2 | 0.1 | No |
| *[Actinomyces] sp.* | Species | Higher in Halitosis | 2.01e-2 | 0.181 | No |
| *Leptotrichia hofstadii* | Species | Higher in Normal | 2.13e-2 | 0.037 | Yes |
| *[Gemella] sp.* | Species | Higher in Halitosis | 3.46e-2 | 0.533 | No |
| *Lautropia mirabilis* | Species | Higher in Normal | 3.66e-2 | 0.245 | No |
| *Campylobacter gracilis* | Species | Higher in Normal | 3.76e-2 | 0.185 | No |
| *Atopobium parvulum* | Species | Higher in Halitosis | 4.31e-2 | 0.25 | No |
| *[Prevotella_7] sp.* | Species | Higher in Halitosis | 4.36e-2 | 0.882 | No |
| *[Anaerovorax] sp.* | Species | Higher in Normal | 4.56e-2 | 0.161 | No |
| *[Rodentibacter] sp.* | Species | Higher in Normal | 4.86e-2 | 0.027 | Yes |
| *Acinetobacter* | Genus | Higher in Normal | 2.36e-15 | 0.003 | Yes |
| *Sphingomonas* | Genus | Higher in Normal | 1.36e-10 | 0.013 | Yes |
| *Haemophilus* | Genus | Higher in Normal | 3.03e-8 | 0.013 | Yes |
| *Mogibacterium* | Genus | Higher in Halitosis | 1.28e-7 | 0.013 | Yes |
| *[Eubacterium] nodatum group* | Genus | Higher in Halitosis | 2.97e-6 | 0.036 | Yes |
| *Prevotella* | Genus | Higher in Halitosis | 7.97e-6 | 0.004 | Yes |
| *Selenomonas* | Genus | Higher in Normal | 3.46e-5 | 0.003 | Yes |
| *Peptostreptococcus* | Genus | Higher in Halitosis | 4.30e-5 | 0.011 | Yes |
| *Suttonella* | Genus | Higher in Normal | 4.30e-5 | 0.012 | Yes |
| *Alloprevotella* | Genus | Higher in Halitosis | 1.02e-4 | 0.003 | Yes |
| *Lautropia* | Genus | Higher in Normal | 1.63e-4 | 0.036 | Yes |
| *Prevotella_7* | Genus | Higher in Halitosis | 2.19e-4 | 0.081 | No |
| *[Eubacterium] yurii group* | Genus | Higher in Normal | 1.93e-3 | 0.064 | No |
| *Dialister* | Genus | Higher in Normal | 3.54e-3 | 0.029 | Yes |
| *Actinobacillus* | Genus | Higher in Normal | 5.45e-3 | 0.142 | No |
| *Johnsonella* | Genus | Higher in Normal | 8.10e-3 | 0.036 | Yes |
| *Solobacterium* | Genus | Higher in Halitosis | 1.53e-2 | 0.308 | No |
| *Actinomyces* | Genus | Higher in Halitosis | 1.65e-2 | 0.166 | No |
| *Neisseria* | Genus | Higher in Normal | 2.30e-2 | 0.02 | Yes |
| *Anaerovorax* | Genus | Higher in Normal | 2.89e-2 | 0.149 | No |
| *Rodentibacter* | Genus | Higher in Normal | 3.03e-2 | 0.023 | Yes |
| *Unclassified* | Genus | Higher in Halitosis | 3.48e-2 | 0.416 | No |
| *Uruburuella* | Genus | Higher in Normal | 3.77e-2 | 0.274 | No |
| *Atopobium* | Genus | Higher in Halitosis | 3.85e-2 | 0.324 | No |

**Table S4. NSTI. PICRUSt2 prediction reliability (weighted Nearest Sequenced Taxon Index) by group.**

Weighted NSTI was calculated as the community-weighted mean NSTI across ASVs retained after excluding those exceeding the default cutoff of 2.0 (14 of 4,598 ASVs excluded genome-wide). Group comparison: Mann-Whitney U test, p = 0.180.

| **Group** | **N** | **Mean NSTI** | **SD** | **Median** | **Min** | **Max** | **N with NSTI > 2.0** |
| --- | --- | --- | --- | --- | --- | --- | --- |
| Halitosis | 45 | 0.0382 | 0.0114 | 0.0339 | 0.0230 | 0.0690 | 0 |
| Normal | 45 | 0.0335 | 0.0048 | 0.0335 | 0.0206 | 0.0425 | 0 |

**Table S5. VSCcorr. Correlation between PICRUSt2-predicted sulfur pathway abundance and measured volatile sulfur compounds.**

Spearman correlations between predicted relative abundance of sulfur-related MetaCyc pathways and directly measured hydrogen sulfide (H₂S) and methyl mercaptan (MM) concentrations (n = 90). Age-adjusted values are partial Spearman correlations controlling for age. The composite score is the sum of the eight pathways listed below, selected from the 14 sulfur-related pathways shown in Fig. 6B based on direct annotation to sulfate assimilation, cysteine/methionine biosynthesis, or heme biosynthesis. Note: HEME-BIOSYNTHESIS-II (heme biosynthesis I, aerobic) shows the opposite direction from the other seven pathways (see Results) but was retained in the composite score because it met the same annotation-based inclusion criterion (direct annotation to heme biosynthesis).

| **Pathway** | **ρ (H₂S)** | **p (H₂S)** | **ρ (H₂S, age-adj.)** | **p (age-adj.)** | **ρ (MM)** | **p (MM)** | **ρ (MM, age-adj.)** | **p (age-adj.)** |
| --- | --- | --- | --- | --- | --- | --- | --- | --- |
| Composite score (sum of 8 pathways) | −0.537 | 4.70e-08 | −0.285 | 0.0068 | −0.641 | 1.04e-11 | −0.404 | 8.55e-05 |
| SULFATE-CYS-PWY (superpathway of sulfate assimilation and cysteine biosynthesis) | −0.458 | 5.72e-06 | −0.196 | 0.0652 | −0.580 | 2.02e-09 | −0.342 | 0.0011 |
| SO4ASSIM-PWY (sulfate reduction I, assimilatory) | −0.475 | 2.18e-06 | −0.210 | 0.0478 | −0.591 | 8.67e-10 | −0.344 | 0.0010 |
| PWY-5345 (superpathway of L-methionine biosynthesis by sulfhydrylation) | −0.484 | 1.32e-06 | −0.219 | 0.0393 | −0.599 | 4.48e-10 | −0.351 | 0.0007 |
| PWY-702 (L-methionine biosynthesis II) | −0.463 | 4.29e-06 | −0.241 | 0.0229 | −0.489 | 1.01e-06 | −0.235 | 0.0263 |
| PWY-724 (superpathway of L-lysine, L-threonine and L-methionine biosynthesis II) | −0.518 | 1.69e-07 | −0.322 | 0.0021 | −0.587 | 1.16e-09 | −0.401 | 9.93e-05 |
| PWY-6641 (superpathway of sulfolactate degradation) | −0.201 | 0.0580 | −0.047 | 0.6631 | −0.234 | 0.0262 | −0.063 | 0.5574 |
| HEME-BIOSYNTHESIS-II (heme biosynthesis I, aerobic) | 0.171 | 0.1065 | −0.037 | 0.7332 | 0.229 | 0.0298 | −0.003 | 0.9750 |
| HEMESYN2-PWY (heme biosynthesis II, anaerobic) | −0.532 | 6.98e-08 | −0.279 | 0.0082 | −0.609 | 1.83e-10 | −0.353 | 0.0007 |

**Table S6. Power. Post-hoc power analysis based on observed effect sizes.**

Statistical power was estimated empirically via subsampling (200–300 iterations per sample size; 999 permutations per PERMANOVA run for the community-level analysis). Power at each sample size was defined as the proportion of iterations yielding p < 0.05.

| **Analysis** | **Observed R² (full sample)** | **Min. n/group for 80% power** | **Min. n/group for 90% power** | **Power at actual n=45/group** |
| --- | --- | --- | --- | --- |
| Community-level (PERMANOVA, Bray-Curtis) | 0.066 | 9 | 10 | 100% |
| Species: *Prevotella_7 melaninogenica* | — | 25 | 30 | 100% |
| Species: *Haemophilus parainfluenzae* | — | 30 | 30 | 100% |
| Species: *Atopobium parvulum* | — | 35 | 40 | 100% |

**Supplementary Figure Legends**

**Fig. S1. Rarefaction curves for all samples.**

Rarefaction curves showing the relationship between sequencing depth and mean observed ASV richness for halitosis (red, n = 45) and healthy control (blue, n = 45) groups. Curves approaching plateau indicate adequate sequencing depth across all samples.

**Fig. S2. Homogeneity of multivariate dispersions.**

Box plot showing the distance to group centroid based on Bray–Curtis dissimilarity for halitosis (n = 45) and healthy control (n = 45) groups. No significant difference in within-group dispersion was detected between groups (permutation test, p = 0.471), confirming homogeneity of dispersions and supporting the validity of PERMANOVA results. ns, not significant. Centre lines indicate medians; box limits indicate the 25th and 75th percentiles; whiskers extend to 1.5× the interquartile range.

**Fig. S3. Beta diversity separation relative to age.**

Principal coordinates analysis (PCoA) plot based on Bray–Curtis dissimilarity, identical to the ordination shown in Fig. 2 (n = 90). (A) Samples coloured by group (halitosis, red circles; healthy control, blue triangles). (B) The same ordination coloured by participant age (years); circles indicate halitosis samples, triangles indicate healthy control samples. PC1 and PC2 were significantly correlated with age (Spearman ρ = −0.24, p = 0.020 and ρ = 0.44, p = 1.4 × 10⁻⁵, respectively), confirming that age contributes to overall community variation; however, halitosis-associated group separation remained visually evident even among age-overlapping individuals.

**Fig. S4. Age-matched sensitivity analysis of beta-diversity group separation.**

(A) Age distribution of halitosis (pink) and healthy control (blue) participants, with the primary age-matched window (22–45 years) shaded. (B) PERMANOVA effect size (R², group effect) for the full sample and four progressively narrower age-overlapping subsets. Group separation remained statistically significant across every window tested, including the tight 27–35-year subset despite its markedly reduced sample size and power (R² = 0.090, p = 0.018, n = 5 halitosis + 15 normal). Exact sample sizes, R², and p-values for each subset are reported in Table S2.

**Fig. S5. Genus-level microbial community composition.**

Stacked bar charts showing the relative abundance (%) of the top 15 genera for each individual sample in the halitosis (left) and healthy control (right) groups. Each colour represents a distinct genus as indicated in the legend. Remaining genera are grouped as 'Other'.

**Fig. S6. Firmicutes/Bacteroidota ratio between halitosis and healthy control groups.**

Box plot showing the log10-transformed Firmicutes/Bacteroidota (F/B) ratio in halitosis and healthy control groups. The halitosis group showed a significantly elevated F/B ratio compared to healthy controls. Statistical significance was assessed by Wilcoxon rank-sum test. ***, p < 0.001. Centre lines indicate medians; box limits indicate the 25th and 75th percentiles; whiskers extend to 1.5× the interquartile range.

**Fig. S7. Species-level microbial community composition.**

Stacked bar charts showing the relative abundance (%) of the top 15 species for each individual sample in the halitosis (left) and healthy control (right) groups. Each colour represents a distinct species as indicated in the legend. Remaining species are grouped as 'Other'.

**Fig. S8. Mean relative abundance of the top 25 species.**

Horizontal bar chart showing the mean relative abundance (%) of the top 25 species across all samples, stratified by group (halitosis, red; healthy control, blue). Species are ranked by overall mean relative abundance.

**Fig. S9. Species-level differential abundance boxplots.**

Box plots showing the relative abundance (%) of the top significantly differentially abundant species between halitosis (red) and healthy control (blue) groups. Statistical significance was determined by Wilcoxon rank-sum test with Benjamini–Hochberg correction. *, adj. p < 0.05; **, adj. p < 0.01; ***, adj. p < 0.001. FDR values are indicated above each panel. Centre lines indicate medians; box limits indicate the 25th and 75th percentiles; whiskers extend to 1.5× the interquartile range.

**Fig. S10. Heatmap of the top 40 differentially abundant MetaCyc pathways.**

Heatmap showing Z-score-normalised predicted relative abundances of the top 40 differentially abundant MetaCyc pathways across all individual samples. Red and blue column bars at the bottom indicate halitosis and healthy control groups, respectively. Colour scale represents Z-score (red: high relative abundance; blue: low relative abundance). Pathways are ordered by differential abundance between groups.

**Fig. S11. Post-hoc power analysis based on observed effect sizes.**

Empirical statistical power estimated by subsampling (200–300 iterations per sample size). (A) Community-level power for the PERMANOVA beta-diversity test (observed R² = 0.066); 80% power was achieved with as few as 9 individuals per group, and power was effectively saturated (100%) at the actual cohort size of 45 individuals per group (dotted line). (B) Species-level power for three representative taxa spanning a range of effect sizes (Atopobium parvulum, Haemophilus parainfluenzae, Prevotella melaninogenica); 80% power was achieved between 25 and 35 individuals per group, with all three taxa reaching 100% power at the actual sample size. Dashed horizontal line indicates 80% power.
